# Mixed pathologies in pancreatic β cells from subjects with neurodegenerative diseases and their interaction with prion protein

**DOI:** 10.1186/s40478-021-01171-0

**Published:** 2021-04-08

**Authors:** Ivan Martinez-Valbuena, Rafael Valenti-Azcarate, Irene Amat-Villegas, Irene Marcilla, Gloria Marti-Andres, Maria-Cristina Caballero, Mario Riverol, María-Teresa Tuñon, Paul E. Fraser, María-Rosario Luquin

**Affiliations:** 1 Neurosciences Division, Center for Applied Medical Research (CIMA), Universidad de Navarra, Pamplona, Spain; 2 Navarra’s Health Research Institute (IDISNA), Pamplona, Spain; 3 Neurology Department, Clinica San Miguel, Pamplona, Spain; 4 Pathology Department, Complejo Hospitalario de Navarra, Pamplona, Spain; 5 Neurology Department, Clinica Universidad de Navarra, Avenida de Pio XII 36, 31008 Pamplona, Navarra Spain; 6 Tanz Center for Neurodegenerative Diseases, University of Toronto, Toronto, Canada

**Keywords:** Alpha-synuclein, Tau, PrP, Type two diabetes mellitus, Parkinson’s disease, Alzheimer’s disease

## Abstract

Protein misfolding diseases refer to a variety of disorders that develop as a consequence of the misfolding of proteins in various organs. The etiologies of Parkinson’s and Alzheimer’s disease remain unclear, but it seems that type two diabetes and other prediabetic states could contribute to the appearance of the sporadic forms of these diseases. In addition to amylin deposition, other amyloidogenic proteins implicated in the pathophysiology of neurodegenerative diseases could have important roles in the pathogenesis of this disease. As we have previously demonstrated the presence of α-synuclein deposits in the pancreas of patients with synucleinopathies, as well as tau and Aβ deposits in the pancreatic tissue of Alzheimer’s disease patients, we studied the immunoreactivity of amylin, tau and α-synuclein in the pancreas of 138 subjects with neurodegenerative diseases or type two diabetes and assessed whether the pancreatic β-cells of these subjects present cooccurrence of misfolded proteins. Furthermore, we also assessed the pancreatic expression of prion protein (PrP) in these subjects and its interaction, both in the pancreas and brain, with α-synuclein, tau, Aβ and amylin. Our study shows, for the first time, that along with amylin, pancreatic α-synuclein, Aβ, PrP and tau may contribute together to the complex pathophysiology of type two diabetes and in the appearance of insulin resistance in Alzheimer’s and Parkinson’s disease. Furthermore, we show that the same mixed pathologies that are observed in the brains of patients with neurodegenerative diseases are also present outside the nervous system. Finally, we provide the first histological evidence of an interaction between PrP and Aβ, α-synuclein, amylin or tau in the pancreas and *locus coeruleus*. These findings will shed more light on the common pathological pathways shared by neurodegenerative diseases and type two diabetes, benefiting the exploration of common therapeutic strategies to prevent or treat these devastating amyloid diseases.

**Supplementary Information:**

The online version contains supplementary material available at 10.1186/s40478-021-01171-0.

## Introduction

Protein misfolding diseases (PMDs) comprise a broad spectrum of conditions, including Alzheimer’s (AD) and Parkinson’s disease (PD), dialysis-related amyloidosis and type two diabetes mellitus (T2DM) [45, 61]. PMDs develop and progress with a distinctive deposition of misfolded proteins such as tau and β-amyloid (Aβ) in AD [8], α-synuclein in PD [19] and dementia with Lewy bodies (DLB) [23], or amylin (also named islet amyloid polypeptide (IAPP)) in T2DM [12]. Although each of these disorders exhibits characteristic protein inclusions, in neurodegenerative diseases several lines of evidence have shown that the cooccurrence of other misfolded proteins related to either other neurodegenerative or nondegenerative (vascular or metabolic) conditions is a frequent event in the brains of these patients [15, 33, 34, 53, 56]. Understanding the impact of these concomitant proteins has an outstanding relevance, as it may help to unravel the considerable differences in clinical presentations of what were previously considered homogenous entities [41].

Furthermore, accumulating evidence from human studies and in vitro and in vivo models indicates that in addition to protein misfolding, intercellular transmission and the subsequent templated amplification of these misfolded proteins are also involved in the onset and progression of these diseases [50]. Although the question is still unresolved, great progress has been made in the identification of novel pathways and molecular partners involved in the uptake and propagation of amyloidogenic proteins. Pinocytosis, endocytosis, diffusion through membranes between cells, transsynaptic connections or tunneling nanotubes [18, 37, 47] are some of the possible mechanisms highlighted for the cellular uptake of these proteins [14, 21, 46, 64]. However, in addition to the mechanisms mentioned above, this uptake can also occur via receptor-mediated endocytosis. In recent years, more than a dozen of receptors have been proposed [6, 17], and among all of them, the prion protein (PrP) has more supportive evidence than most [17, 59]. Recently, several studies have suggested a role for PrP in binding to and mediating α-synuclein, Aβ and tau toxicity [20, 28, 48]. PrP is a glycoprotein tethered to the plasma membrane via a glycosylphosphatidylinositol anchor [68], and it is best known for its role in the pathogenesis of prion diseases [51]. PrP is mainly expressed in the central nervous system as well as in the pancreatic islets, spleen, testes and other organs [13, 63]. Although still elusive and to some extent controversial, PrP seems to play an important role in several cellular functions, including the self-renewal of long-term repopulation of hematopoietic stem cells [68, 72]. Furthermore, some studies also point to an important role of PrP in endocrine function [3, 4, 11, 63]. In fact, PrP knockout mice have impaired glucose tolerance [63], and a direct relationship between PrP and insulin response has been suggested since PrP is overexpressed upon insulin treatment [35]. As our group previously demonstrated the presence of phosphorylated forms of α-synuclein in the pancreatic tissue of patients with synucleinopathies [39], as well as deposition of phosphorylated, truncated and oligomeric forms of tau and of Aβ in the pancreatic tissue of AD patients [40], we studied whether pancreatic β-cells of subjects with neurodegenerative diseases present cooccurrence of other misfolded proteins, as observed in the brain. Furthermore, we also assessed the pancreatic expression of PrP in subjects with AD, PD, DLB or T2DM and its interaction, both in the pancreas and brain, with α-synuclein, tau, Aβ and amylin.

## Methods

### Study design and participants

We carried out a retrospective study based on whole-body autopsies performed between 2000 and 2017 at the Pathology Service of the Complejo Hospitalario de Navarra (Spain). All subjects included in the study received a detailed neuropathological examination, and they were distributed into different groups according to their neuropathological postmortem diagnosis. Specific consensus diagnostic criteria for PD [1, 9] DLB [9, 42], incidental Lewy body disease (ILBD) [7] and AD [29, 43] were used to establish the neuropathological diagnosis. Accordingly, we included 10 subjects with neuropathological findings compatible with PD and 12 that met the diagnostic criteria for DLB. A similar number of subjects diagnosed with ILBD were included (n = 8). Furthermore, we included 62 subjects with neuropathological findings compatible with AD, of whom 28 subjects met the diagnostic criteria for AD with low neuropathologic changes, 26 AD subjects exhibited intermediate neuropathologic changes and eight subjects met the diagnostic criteria for AD with high neuropathologic changes. Subjects with a neurodegenerative disease and T2DM antecedent were excluded. Furthermore, 46 aged-matched control subjects with no neuropathological abnormalities were also included. These subjects were distributed into two groups according to the clinical diagnosis of T2DM, leaving one group of 19 subjects with a normal neuropathological examination and a history of T2DM and another of 27 control subjects with a normal neuropathological examination and no history of T2DM. Written informed consent for whole-body autopsy and the removal of all organs for diagnostic and research purposes was obtained for all the subjects from their next of kin.

### Procedures

#### Tissue dissection and preparation

Morphological studies were performed on one formalin-fixed (10%) hemisphere (usually the right) and after fixation (21–25 days), representative tissues from distinct brain areas were embedded in paraffin to perform the neuropathological analysis on 4 µm sections from these areas: gyrus frontalis medius, gyrus temporalis superior and medius, gyrus parietalis inferior, gyrus hippocampi anterior (including *Cornu Ammonis* (CA) regions I-IV and entorhinal cortex), basal forebrain (including amygdala, nucleus of Meynert), central gray matter (including globus pallidus, putamen, caudate, thalamus and nucleus subthalami), substantia nigra, pons (including *locus coeruleus* and Raphe nucleus), motor nucleus of vagus, cerebellum (including vermis cerebelli, nucleus dentatus and cortex), and spinal cord. To examine the pancreas, it was immediately immersed in 10% formalin upon removal and fixed for a median of 24 h (range 20–72 h), and after macroscopic examination, part of this organ was embedded in paraffin to obtain 4 µm sections for analysis.

#### Immunohistochemistry

Immunohistochemical examination of the brain was performed with a DAKO Autostainer (DAKO, Glostrup, Denmark). The sections were stained using mouse monoclonal antibodies against phosphorylated tau (Novocastra, NCL-Tau-2, clone Tau-2, diluted 1:100), α-synuclein (Novocastra, NCL-L-ASYN, clone KN51, diluted 1:50) and β-amyloid (Novocastra, NCL-β-amyloid, clone 6F-3D, diluted 1:200). Antibody binding was detected with a biotinylated secondary antibody (goat anti-mouse), and the antibodies were visualized using an avidin–biotin–peroxidase complex with 3·3′diaminobenzidine tetrahydrochloride (DAB) as the chromogen. To examine the pancreatic tissue, immunohistochemistry was performed using a panel of different, previously validated [39, 40], antibodies against amylin (Abcam, ab15125, diluted 1:100; Abcam, ab77387, diluted 1:50), tau (Dako, A0024, diluted 1:100; Abcam, ab32057, diluted 1:3000) or α-synuclein (Millipore, MAB5320, diluted 1:1000; Proteintech, 10842-1-AP, diluted 1:500). For PrP immunohistochemistry, two antibodies were used (SpiBio, clone 12F10, diluted 1:500 and Abcam, ab6664, diluted 1:200). As a positive control for PrP staining, we performed immunohistochemistry of both antibodies on tissue sections containing the cerebellum of a Creutzfeldt-Jakob disease patient. Moreover, to ensure that the immunoreactivity in the samples was not due to cross-reactivity or a nonspecific reaction of the antibodies in the pancreatic tissue, one of the antibodies (ab6664) was incubated overnight at 4 °C with its blocking peptide (1 μg/ml of human Prion Protein peptide, ab49415, Abcam). Immunohistochemistry with the PrP^c^ antibody blocked with the peptide was performed under the same conditions on ten pancreatic sections. These slides were analyzed blindly, and all samples incubated with the blocked antibody were classified as negative for PrP^c^ staining. To test whether PrP^c^-immunoreactive pancreatic sections contain proteinase K-resistant PrP aggregates, we treated pancreatic sections with proteinase K (Proteinase K-ready to use, Dako) for 2 min before staining with anti-PrP antisera (12F10) as described above.

In addition, to assess α-synuclein posttranslational modifications present in the pancreatic tissue, two monoclonal antibodies against serine 129 phosphorylated α-synuclein (Wako, clone psyn#64, diluted 1:2,000 and Abcam, ab209422, diluted 1:500) were used. These antibodies are specific for and sensitive to phosphorylated α-synuclein in pathological structures [22], and they have been used previously to study inclusions in peripheral tissues [39, 66]. Furthermore, we also used antibodies against nitrated (Biolegend, clone 514, diluted 1:500) and C-terminal truncated α-synuclein (Biolegend, clone A15127A, diluted 1:500). As positive controls, we performed immunohistochemistry on tissue sections containing the substantia nigra of two PD patients. For the assessment of tau conformational and posttranslational modifications, we performed single immunohistochemistry against AT8 (phospho-tau Ser202-Thr205, Thermo Fisher, MN1020, diluted 1:100), AT100 (Thr212-Ser214 phospho-tau, ThermoFisher, MN1060, diluted 1:200), AT180 (Thr231 phospho-tau, ThermoFisher, MN1040, diluted 1:200), AT270 (Thr181 phospho-tau, ThermoFisher, MN1050, diluted 1:250), Ser422 phospho-tau (ThermoFisher, 44-764G, diluted 1:250), Ser262 phospho-tau (ThermoFisher, 44-750G, diluted 1:250), Asp421 cleaved tau (Merck, clone C3, diluted 1:250), tau Alz50 (a generous gift from Dr Peter Davies, Feinstein Institute for Medical Research, Manhasset, NY, diluted 1:100diluted 1:100), tau MC-1 (a generous gift from Dr Peter Davies, diluted 1:100), oligomeric tau (Merck, clone T22, diluted 1:750) and Aβ (clone 4G8, diluted 1:100). As positive controls, we performed immunohistochemistry for all the aforementioned antibodies on tissue sections containing the hippocampus of an AD patient.

As pancreatic tissue is very prone to autolysis and antibodies could bind nonspecifically to secretory proteins or enzymes, we also stained the gastric mucosa (for secretory cell internal control) for all the antibodies used to assess that the immunoreactivities observed in the pancreatic tissue were specific (Additional file 1: Fig. S1). Antibody binding was detected using the EnVision + System-HRP labeled polymer (Dako) with DAB as the chromogen. After immunostaining, the sections were counterstained with hematoxylin–eosin.

#### Immunoreactivity quantification

Immunohistochemistry against amylin, tau, PrP and α-synuclein was quantified using an Olympus BX-51 microscope equipped with an Olympus DP-70 camera using the CAST grid software package (Olympus). We quantified the immunoreactivity obtained against each protein described above in approximately 15 islets of Langerhans for each subject. Once obtained, the images were color-deconvoluted using ImageJ software (NIH, Bethesda, USA), as previously described [57]. Then, the perimeter of each islet of Langerhans was outlined manually for each image excluding any unwanted immunostained structures (i.e., capillary), and the immunoreactive area was determined. To minimize the caveats of this method and avoid variability between slides, we performed immunohistochemistry for one marker in consecutive rounds to ensure that the same batch of the antibody and the same reagents were used. Furthermore, we exposed all the sections to DAB for two minutes, and the same investigator performed all quantifications with masked sections.

For the rest of the antisera used, immunoreactivity was assessed for the whole slide using a semiquantitative score by two independent observers. Protein immunoreactivity was examined in pancreatic tissue using a conventional light microscope (BX51, Olympus) at low magnification and scored as 0: no staining; 1: weak staining, 2: moderate staining, and 3: strong staining. Scores 1, 2 and 3 were considered positive, and score 0 was considered negative (Additional file 1: Fig. S2). Furthermore, we considered a subject positive or negative for protein inclusions when there was full agreement between the two assessments.

#### Immunofluorescence

Pancreatic sections were stained overnight at 4 °C, combining PrP (12F10 or ab6664), tau (A0024) and Aβ (4G8) antibodies with the following antibodies: insulin (Abcam, ab7842, diluted 1:100), somatostatin (Abcam, ab30788, diluted 1:50) or glucagon (Abcam, ab10988, diluted 1:1,000), and combining Aβ primary antibody (4G8) with the anti-oligomer A11 antibody (ThermoFisher, AHB0052, diluted 1:1,000). After washing in phosphate-buffered saline (PBS), the sections were incubated for 2 h at room temperature with Alexa Fluor 488- or 564-conjugated secondary antibodies (Invitrogen, diluted 1:500) and washed again with PBS and diethyl-pyrocarbonate water. An autofluorescence elimination step was performed using the autofluorescence eliminator reagent (EMD Millipore, 2160), following the manufacturer’s instructions, and then the slides were covered with mounting medium containing DAPI (Sigma, DUO82040). Furthermore, the appropriate negative controls were included to ensure immunofluorescence specificity. For thioflavin S experiments, pancreatic sections were immersed for 8 min with 0.05% thioflavin S (Sigma) and then washed 3 times with 80% ethanol for 5 min, followed by 2 washes in PBS for 5 min. The sections were then incubated with an antibody against serine 129 phosphorylated α-synuclein (Psyn#64) or against tau (A0024), washed 3 times with PBS, and then incubated with an Alexa Fluor 564-conjugated secondary antibody (Invitrogen, diluted 1:500). For double immunofluorescence, an autofluorescence elimination step was performed according to the manufacturer’s protocol. Afterward, the slides were cover-slipped in mounting medium for microscopy. Colocalization images were obtained on a confocal microscope (Zeiss LSM 800 with Airyscan; confocal super-resolution imaging).

#### Proximity ligation assays (PLA)

We performed proximity ligation assays (PLA) to assess whether α-synuclein oligomers were present in pancreatic β cells and whether there was a direct interaction between PrP and α-synuclein, tau, Aβ or amylin both in pancreatic and brain tissue. For the α-synuclein PLA, we used the Duolink in situ Red PLA detection kit (Sigma, DUO92101), according to the manufacturer’s instructions and as described previously [58]. For this assay, the same mouse monoclonal α-synuclein antibody (Abcam, syn211, ab80627) was used to make both probes. To assess whether there was a direct interaction between PrP and Aβ, α-synuclein, tau or amylin on pancreatic and brain sections, the experiments were also carried out using the Duolink in situ Red PLA detection kit (Sigma, DUO92101), as described previously [39, 40]. We performed 8 different PLAs using combinations of PrP (12F10) with some of the primary antibodies used for the immunohistochemical studies (see above): α-synuclein (MAB5320), serine 129 phosphorylated α-synuclein (pSyn#64), tau (A0024), oligomeric tau (clone T22), Aβ (clone 4G8), Aβ_1-42_ (BioLegend, clone 12F4, diluted 1:2,500) and amylin (ab15125 for pancreatic PLA and a rabbit-polyclonal antisera, Bachem Peninsula Laboratories, diluted 1:250 for brain PLA). Furthermore, the appropriate negative PLA controls were included, including a PLA of PrP (12F10) with chromogranin A (CgA, Abcam, ab15160, diluted 1:250), to ensure PLA specificity in all the assays performed (Additional file 1: Figs. S1 and S3). To identify the pancreatic cells where this specific protein–protein interaction occurred, immunofluorescent staining for insulin was performed after carrying out the PLAs using a guinea-pig polyclonal antiserum (Abcam, ab7842, diluted 1:100) to localize the pancreatic β cells. PLA images were obtained on a confocal microscope (Zeiss LSM 800 with Airyscan; confocal super-resolution imaging).

#### Statistical analysis

Statistical analysis was conducted using Prism 8.3 (GraphPad Software Inc.). Data are expressed as the median (25th/75th percentiles) or number (percentage) as appropriate. We performed a Spearman correlation and a Kruskal–Wallis test corrected for multiple comparisons (Bonferroni’s method) to confirm the independence between the number of pancreatic islets studied per patient and the clinical diagnosis. Data were tested for normal distribution using a D’Agostino-Pearson test. To compare the means of pancreatic islet immunoreactivity between clinical diagnosis groups, a Kruskal–Wallis test corrected by multiple comparisons (Bonferroni’s method) was used. Categorical variables were analyzed using Fisher’s exact test.

## Results

Pancreatic tissue from 138 autopsies was evaluated in this retrospective study. After the initial analysis of hematoxylin–eosin-stained pancreatic tissue to confirm the presence of endocrine and exocrine glandular tissue in all the samples, subjects were characterized as follows: individuals with no neuropathologic alterations (n = 46), among whom 19 subjects had a history of T2DM and 27 subjects did not (control subjects); individuals with an AD diagnosis and low neuropathologic changes (n = 28); individuals with an AD diagnosis and intermediate neuropathologic changes (n = 26); and eight individuals with an AD diagnosis and high neuropathologic changes. Furthermore, we included 10 subjects with neuropathological findings compatible with PD and 12 that met the diagnostic criteria of DLB. A similar number of subjects diagnosed with ILBD were included (n = 8). Subjects with a neurodegenerative disease and history of T2DM were excluded. The demographic and neuropathological features of the subjects analyzed are summarized in Table 1 and listed in Additional file 1: Tables S1 and S2.

**Table 1:** General demographic, clinical and neuropathological characteristics of the subjects included in this study

Groups	Number of cases	Male/Female	Age (years)	PMD (hours)
Subjects with a normal neuropathological examination	27	19/8	70 (65–75)	6 (3–8)
Subjects with a normal neuropathological examination and T2DM	19	11/8	lg	6 (4–8)
AD with low neuropathologic change	28	14/14	77 (73–81)	6 (4–8)
AD with intermediate neuropathologic change	26	11/15	78 (75–82)	5 (4–6)
AD with high neuropathologic change	8	3/5	83 (76–92)	5 (3–6)
Incidental Lewy body disease	8	7/1	71 (66–76)	7 (6–8)
Parkinson's disease	10	5/5	77 (74–82)	6 (5–8)
Dementia with Lewy bodies	12	9/3	82 (76–88)	6 (5–8)

The data are the median (25th/75th percentiles) or n (%): PD, Parkinson’s disease; DLB, dementia with Lewy bodies; ILBD, incidental Lewy body disease; AD, Alzheimer’s disease; T2DM, type two diabetes mellitus; PMD, postmortem delay

### Amylin, α-synuclein, tau and PrP immunoreactivity is increased in pancreatic cells of subjects with neurodegenerative diseases or T2DM

**Fig. 1:**
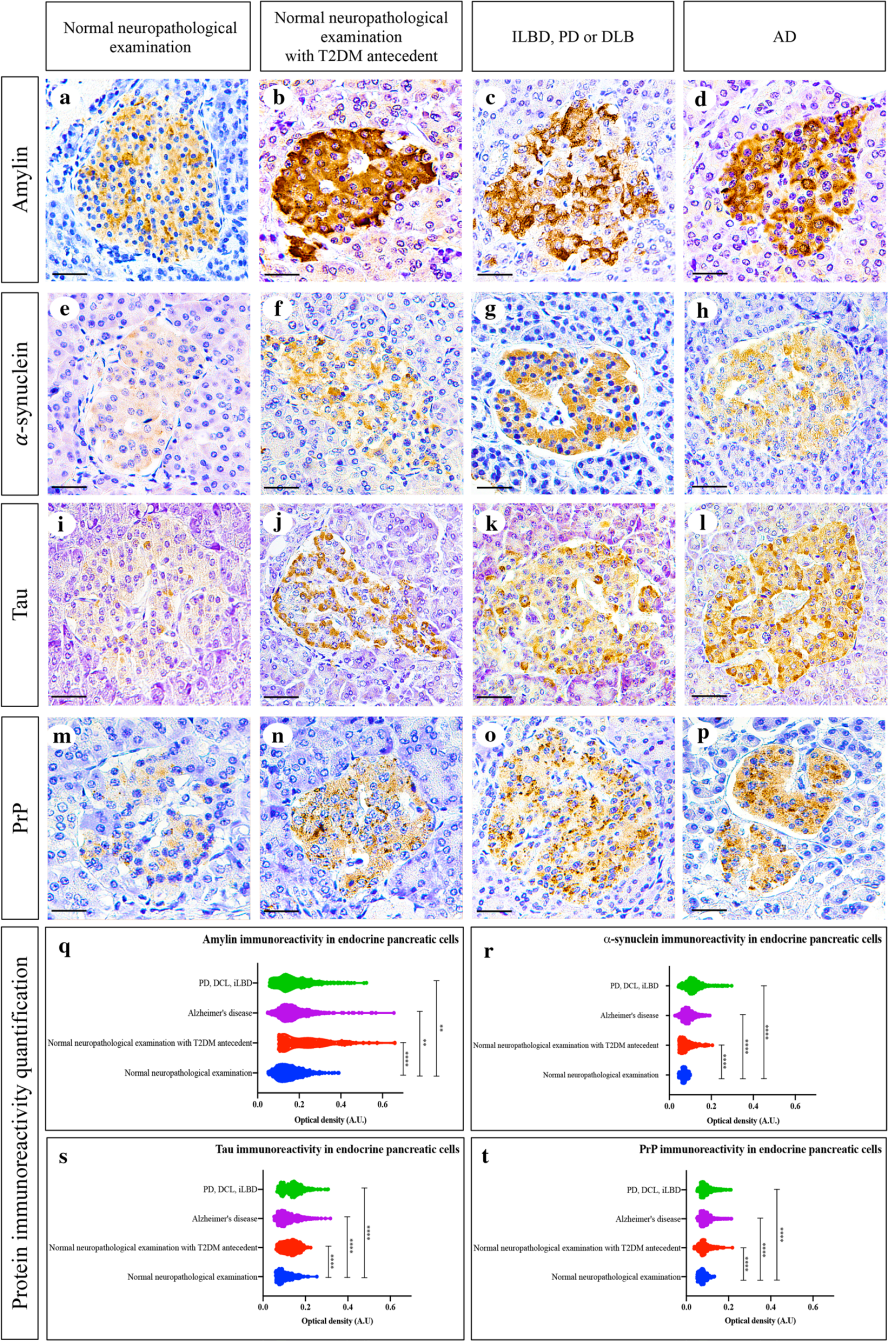
Amylin, α-synuclein, tau and PrP immunoreactivity in endocrine pancreatic cells. Immunohistochemistry for amylin (**a**–**d**), α-synuclein (**e**–**h**), Tau (**i**–**l**), and PrP (**m**–**p**) in pancreatic cells from subjects with a normal neuropathological examination and no history of T2DM (**a**, **c**, **i**, **m**), with a normal neuropathological examination and history of T2DM (**b**, **f**, **j**, **n**), with Parkinson`s disease (PD), dementia with Lewy bodies (DLB) or incidental Lewy body disease (ILBD) (**c**, **g**, **k**, **o**) or with Alzheimer’s disease (AD) (**d**, **h**, **l**, **p**): 40 × magnification; scale bar = 50 µm. Violin plots showing amylin (**q**), α-synuclein (**r**), Tau (**s**) and PrP^c^ (**t**) immunoreactivity quantification in endocrine pancreatic cells: **: *p* ≤ 0.05, ***: *p* ≤ 0.001; ****: *p* ≤ 0.0001

Amylin, α-synuclein, tau and PrP were expressed widely in the cytoplasm of endocrine pancreatic cells (Fig. 1a–p and Additional file 1: Fig. S4), and a semiquantitative analysis of these proteins identified marked differences in the immunoreactivity between the different groups of subjects included. Amylin had the most prominent immunoreactivity in pancreatic β cells and as expected, it was more abundant in subjects with a normal neuropathological examination and a history of T2DM than in controls (*p* < 0.0001). When amylin immunoreactivity was assessed in subjects with a synucleinopathy or with AD, we found increased immunoreactivity of amylin compared to that in controls (*p* = 0.0085 for synucleinopathies, *p* = 0.0011 for AD) but lower immunoreactivity than in subjects with a normal neuropathological examination and a history of T2DM (*p* < 0.0001 for synucleinopathies, *p* < 0.0001 for AD). We did not find differences in amylin immunoreactivity between patients with neurodegenerative diseases (*p* > 0.9999, Fig. 1q). Likewise, α-synuclein was more immunoreactive in subjects with a normal neuropathological examination and a history of T2DM than in controls (*p* < 0.0001). However, subjects with synucleinopathies had higher α-synuclein pancreatic immunoreactivity than controls (*p* < 0.0001), subjects with a normal neuropathological examination and a history of T2DM (*p* < 0.0001) or subjects with AD (*p* < 0.0001, Fig. 1r). When tau expression was assessed, we found prominent pancreatic tau immunoreactivity in subjects with a normal neuropathological examination and a history of T2DM compared with controls (*p* < 0.0001). However, subjects with AD had higher pancreatic tau immunoreactivity than controls (*p* < 0.0001), subjects with a normal neuropathological examination and a history of T2DM (*p* < 0.0001) or subjects with synucleinopathy (*p* < 0.0001). A wider pancreatic tau immunoreactivity was found in patients with synucleinopathies than in controls (*p* < 0.0001), but we did not find differences in pancreatic tau between subjects with synucleinopathies and subjects with normal neuropathological examination and a history of T2DM (*p* = 0.2323, Fig. 1s). PrP pancreatic immunoreactivity was more abundant in subjects with a normal neuropathological examination and a history of T2DM than in controls (*p* < 0.0001). However, subjects with synucleinopathies or with AD presented a wider PrP immunoreactivity than subjects with a normal neuropathological examination and a history of T2DM (*p* < 0.0001 for synucleinopathies and *p* = 0.0012 for AD subjects) or controls (*p* < 0.0001 for synucleinopathies and AD subjects). When differences between AD subjects and patients with synucleinopathies were assessed, we did not find differences in pancreatic PrP immunoreactivity (*p* = 0.1338, Fig. 1t).

### Different forms of α-synuclein are found in pancreatic β-cells of subjects with neurodegenerative diseases or with T2DM

**Fig. 2:**
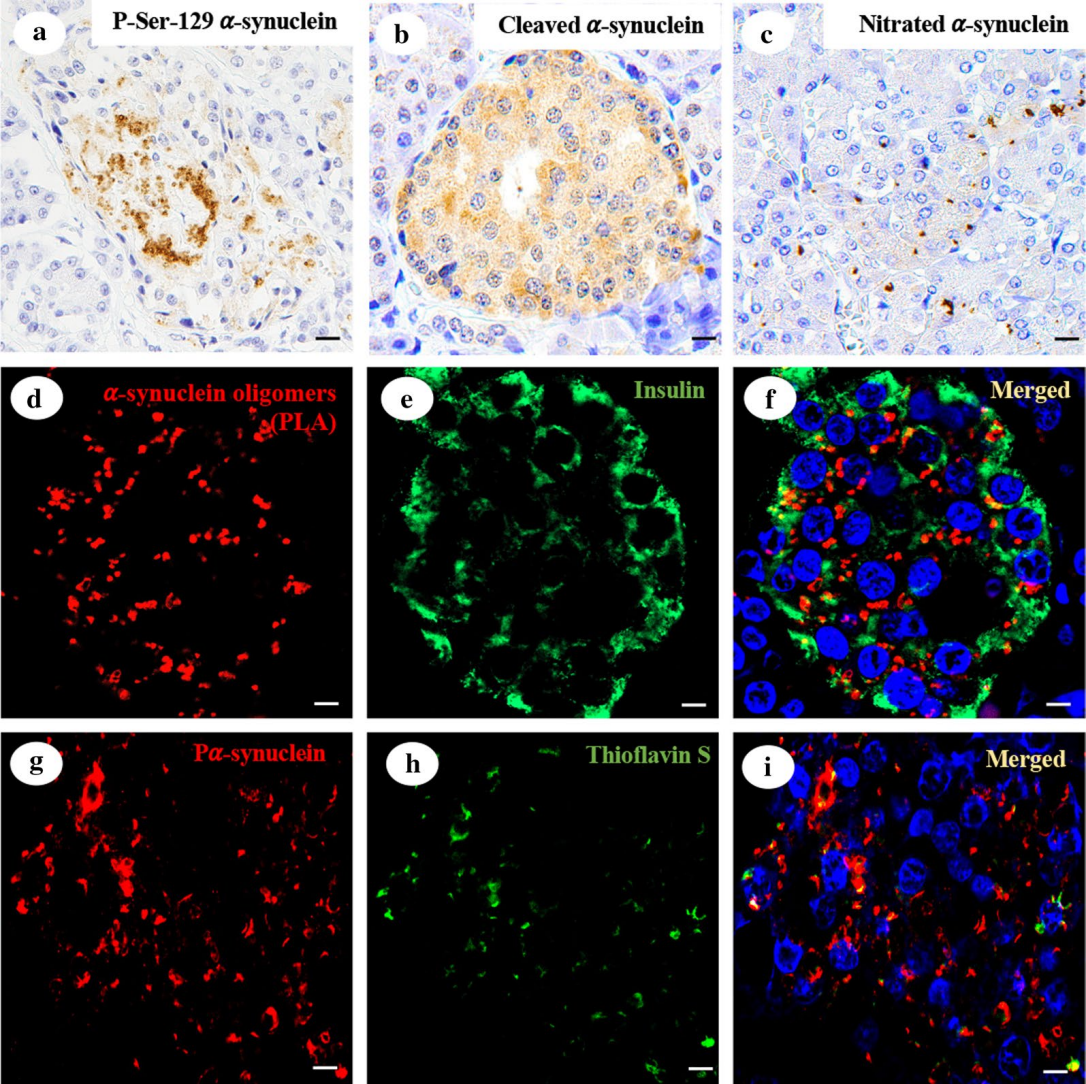
α-synuclein deposition in pancreatic β cells of subjects with Alzheimer’s disease. Immunohistochemistry for serine 129-phosphorylated α-synuclein immunofluorescence (**a**) C-terminal cleaved α-synuclein (**b**) and nitrated α-synuclein (**c**) from a 70-year-old female with Alzheimer’s disease. α-synuclein PLA (**d**), with insulin immunofluorescence (**e**) and DAPI as nuclear counterstaining (**f**) from a 73-year-old female with Alzheimer’s disease. Serine 129-phosphorylated α-synuclein immunofluorescence (**g**) and thioflavin S fluorescence (**h**) in pancreatic β cells from a 77-year-old female with Alzheimer’s disease (**i**): 40 × magnification; scale bar = 50 µm

Cytoplasmic phosphorylated α-synuclein expression was found in pancreatic β cells from 11 subjects with AD and low neuropathologic change (39%), 13 subjects with AD and intermediate neuropathologic change (50%) and six subjects with AD and high neuropathologic change (75%, Fig. 2a, summarized in Table 2 and listed in Additional file 1: Table S3). Thus, subjects with AD had more pancreatic phosphorylated α-synuclein deposits than controls (*p* = 0.0095) but fewer deposits than subjects with synucleinopathies (*p* = 0.0015). Furthermore, we did not find differences in phosphorylated α-synuclein pancreatic deposition between patients with AD and subjects with a normal neuropathological examination and a history of T2DM (*p* = 0.188).

**Table 2:** Subjects with AD, PD, DLB, ILBD and with a normal neuropathological examination exhibited α-synuclein deposits in pancreatic β cells

Groups	Pα-synuclein	Cleaved α-synuclein	Nitrated α-synuclein
Subjects with a normal neuropathological examination	5/27 (19%)*	13/27 (48%)	4/27 (15%)
Subjects with a normal neuropathological examination and T2DM	6/13 (68%)*	18/19 (95%)	7/19 (37%)
AD with low neuropathologic change	11/28 (39%)	23/28 (82%)	7/28 (25%)
AD with intermediate neuropathologic change	13/26 (50%)	25/26 (96%)	15/26 (58%)
AD with high neuropathologic change	6/8 (75%)	8/8 (100)	4/8(50%)
Incidental Lewy body disease	5/8 (63%)*	8/8 (100%)	3/8 (38%)
Parkinson's disease	9/10 (90%)*	10/10 (100%)	6/10 (60%)
Dementia with Lewy bodies	11/12 (91%)*	12/12 (100%)	6/12 (50%)

The data are represented as n (%). AD, Alzheimer disease; PD, Parkinson’s disease; DLB, dementia with Lewy bodies; ILBD, incidental Lewy body disease; T2DM, type two diabetes mellitus. *These results had been previously published in Martinez-Valbuena, et al. 2018 and have been added in this table to facilitate comparisons between groups

Cytoplasmic truncated α-synuclein was widely found in more than 80% of subjects with AD, in 95% of subjects with a normal neuropathological examination and a history of T2DM and in all the subjects with a synucleinopathy (Fig. 2b). This expression was higher than that in control subjects, where 13 subjects (48%) presented pancreatic cytoplasmic truncated α-synuclein expression (*p* = 0.0011 for T2DM subjects, *p* < 0.0001 for synucleinopathies and AD subjects, summarized in Table 2 and listed in Additional file 1: Table S3). In addition, cytoplasmic nitrated α-synuclein expression was found in pancreatic β cells from seven subjects with AD and low neuropathologic change (25%), 15 subjects with AD and intermediate neuropathologic change (58%) and four subjects with AD and high neuropathologic change (50%). Furthermore, nitrated α-synuclein expression was detected in 3 subjects with ILBD (38%), 6 subjects with PD (60%), 6 subjects with DLB (50%), 7 subjects considered to be normal following neuropathological examination but with T2DM (37%) and 4 control subjects (15%, Fig. 1c, summarized in Table 2 and listed in Additional file 1: Table S3). To assess whether α-synuclein oligomers were present in the pancreatic β cells of the subjects, we performed a specific PLA for α-synuclein oligomers. First, we assessed the specificity of the assay in our experimental conditions, and we performed the PLA technique in sections of the substantia nigra of two control subjects and two patients with PD. We did not obtain a PLA signal in controls, while we obtained a dot-like pattern in the neuronal cell bodies of PD patients. Once optimized, the assay was performed on pancreatic samples. We obtained a cytoplasmic dot-like pattern circumscribed to pancreatic β cells (Fig. 2d–f), confirming the presence of α-synuclein oligomers in the pancreas of subjects with AD, synucleinopathies and subjects with a normal neuropathological examination and a history of T2DM. Furthermore, we also found a specific PLA signal in three of the five control subjects who had phosphorylated α-synuclein pancreatic inclusions. Finally, to determine whether in addition to oligomeric species, the cytoplasmic α-synuclein detected in the pancreatic β cells of subjects with neurodegenerative diseases or T2DM was aggregated, we performed thioflavin S staining, which binds specifically to amyloid fibrils. This was assessed immediately after phosphorylated α-synuclein immunofluorescence to determine whether the amyloid fibrils that were stained corresponded to phosphorylated α-synuclein deposits. Thioflavin S colocalized with phosphorylated α-synuclein, indicating that pancreatic α-synuclein is aggregated (Fig. 2g–i).

### Tau and Aβ deposits are found in pancreatic β-cells of subjects with synucleinopathies

**Fig. 3:**
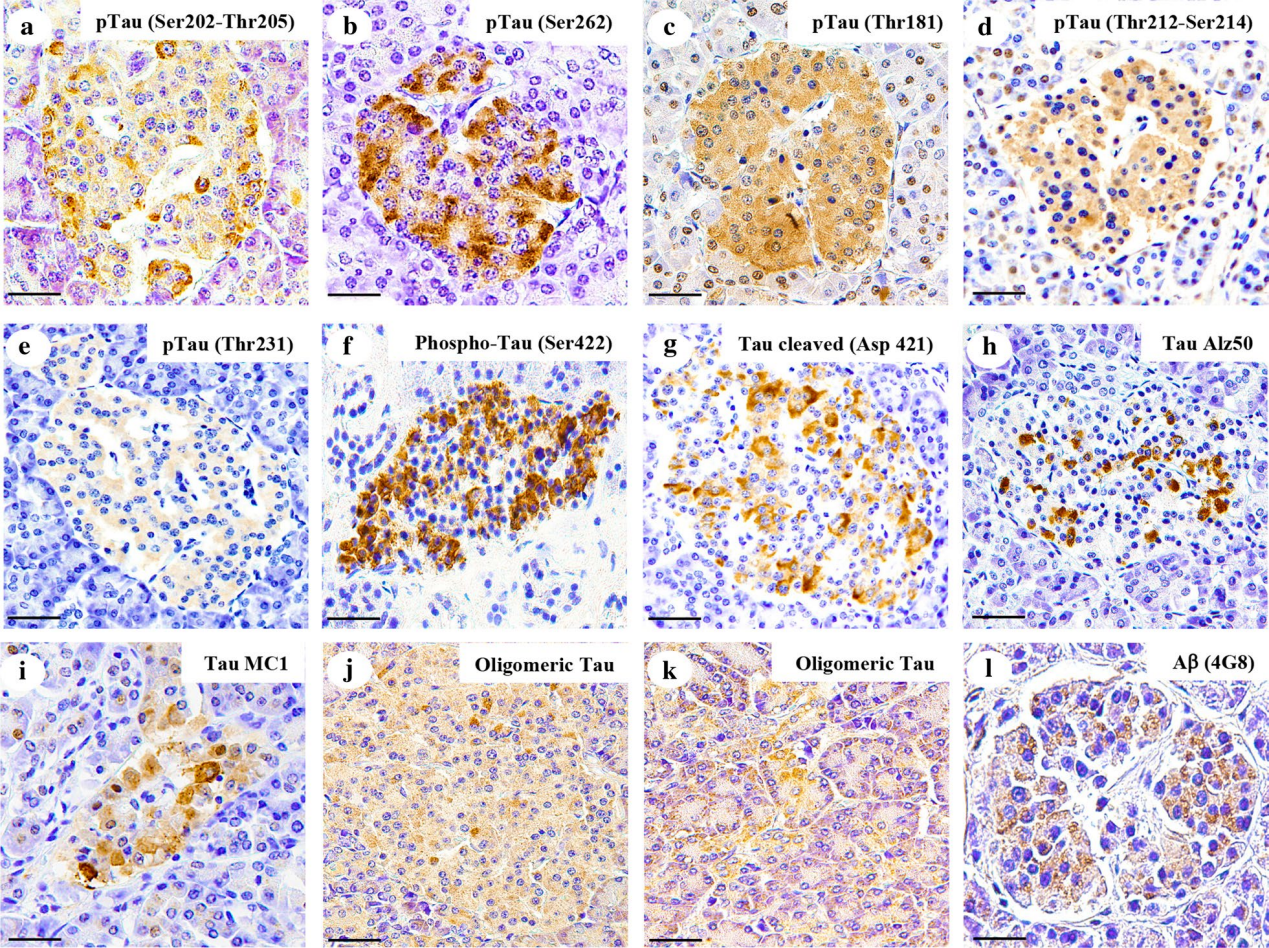
Tau and Aβ inclusions in pancreatic β cells of subjects with Parkinson’s disease, Lewy body dementia and incidental Lewy body disease. Immunohistochemistry for Ser202-Thr205-phosphorylated tau (AT8) (**a**), Ser262-phosphorylated tau (**b**), Thr181-phosphorylated tau (AT270) (**c**), Thr212-Ser214-phosphorylated tau (AT100) (**d**), Thr231-phosphorylated tau (AT180) (**e**), Ser422-phosphorylated tau (**f**), Asp421-cleaved tau (**g**), tau Alz50 (**h**), tau MC-1 (**i**), endocrine oligomeric tau (**j**), exocrine oligomeric tau (**k**) and Aβ (**l**) inclusions in pancreatic cells from subjects with incidental Lewy body disease (**a**, **b**, **c**, **f**), Parkinson’s disease (**d**, **e**, **g**, **h**, **i**, **l**) and dementia with Lewy body (**j**, **k**): a 40 × magnification; scale bar = 50 µm

To evaluate the pancreatic expression of phosphorylated tau in subjects with synucleinopathies, we examined immunohistochemical staining with six antibodies that specifically recognize tau phosphorylated at residues Ser202-Thr205 (AT8), Thr212-Ser214 (AT100), Thr231 (AT180), Thr181 (AT-270), Ser422 and Ser262. Cytoplasmic expression of Thr181- and Ser262-phosphorylated tau (Fig. 1b–c, summarized in Table 3 and listed in Additional file 1: Table S4) was evident in more than 80% of the subjects, irrespective of their neuropathologic diagnosis. In turn, the expression of tau phosphorylated at Ser202-Thr205, Thr212-Ser214, Thr231 and Ser422 (Fig. 1a, d–f) was more enhanced in subjects with synucleinopathies than in controls (*p* = 0.003 for Ser202-Thr205, *p* = 0.0025 for Thr212-Ser214, *p* = 0.0424 for Thr231 and *p* = 0.0316 for Ser422) and in subjects with a normal neuropathological examination and a history of T2DM (*p* = 0.0101 for Ser202-Thr205, *p* = 0.0055 for Thr212-Ser214, *p* = 0.0456 for Thr231 and *p* = 0.0316 for Ser422). However, no differences were found in the expression of these markers between patients with neurodegenerative diseases. Likewise, when the expression of Asp421-cleaved tau was assessed (Fig. 3g), we found more subjects with synucleinopathies and pancreatic truncated tau deposits than subjects with a normal neuropathological examination with (*p* = 0.0010) or without (*p* < 0.0001) T2DM history. We also used the Alz50 and MC1 conformational antibodies and found Alz50 in pancreatic β-cells from more than 75% of the subjects (Fig. 3h, summarized in Table 3 and listed in Additional file 1: Table S4). Conversely, only two subjects with ILBD (25%), four subjects with PD (40%) and five with DLB (42%) were MC1 positive, whereas 88% of subjects with AD and high neuropathologic changes showed MC1 positive deposits (*p* = 0.0162, Fig. 3i, summarized in Table 3 and listed in Additional file 1: Table S4). Oligomeric tau expression was assessed with the T22 antibody, and it was detected in more than 80% of subjects with synucleinopathies, both in endocrine and exocrine pancreatic tissue. Compared to control subjects, only seven subjects presented immunoreactivity against the T22 antibody (*p* = 0.0002, Fig. 3j–k and Additional file 1: Fig. S5). Finally, we performed thioflavin S staining to assess if the cytoplasmic tau detected in the pancreatic β cells of subjects with neurodegenerative diseases or T2DM was aggregated. The thioflavin S staining colocalized with tau, indicating that pancreatic tau is aggregated (Additional file 1: Fig. S6). When pancreatic Aβ expression was assessed, immunoreactivity similar to that observed in diffuse brain Aβ plaques (Fig. 3l) was apparent in nine subjects with synucleinopathies (30%, summarized in Table 3 and listed in Additional file 1: Table S4). To assess whether Aβ oligomers were present in the pancreatic β cells of the subjects, we performed double immunofluorescence against Aβ and the oligomeric A11 antibody, and we observed a co-localization between these two markers, indicating that these patients have Aβ oligomers in the pancreatic β-cells (Additional file 1: Fig. S7). Furthermore, we assessed that the immunoreactivity shown by all subjects included was specific for pancreatic tissue, since we did not observe tau or Aβ immunoreactivity in the gastric mucosa (Additional file 1: Fig. S1).

**Table 3:** Subjects with PD, DLB and ILBD exhibit specific tau and Aβ deposits in pancreatic β cells

Groups	pSer202-Thr205	pThr212-Ser214	pThr231	pThr181	pSer262 Tau	pSer 422 Tau	Oligomeric Tau	Asp421-cleaved Tau	Tau	Tau	Aβ (4G8)
	Tau	Tau	Tau	Tau					Alz50	MC1	
Incidental Lewy body disease	7/8 (88%)	6/8 (75%)	1/8 (13%)	8/8 (100%)	8/8 (100%)	5/8 (63%)	10/10 (100%)	6/8 (75%)	6/8 (75%)	2/8 (25%)	2/8 (25%)
Parkinson's disease	10/10 (100%)	9/10 (90%)	4/10 (40%)	10/10 (100%)	10/10 (100%)	8/10 (80%)	8/10 (80%)	8/10 (80%)	10/10 (100%)	4/10 (40%)	3/10 (30%)
Dementia with Lewy bodies	12/12 (100%)	12/12 (100%)	7/12 (58%)	12/12 (100%)	12/12 (100%)	10/12 (83%)	9/12 (83%)	10/12 (83%)	12/12 (100%)	5/12 (42%)	4/12 (33%)
Normal neuropathological examination*	15/29 (52%)	14/29 (48%)	7/29 (24%)	24/29 (83%)	26/29 (90%)	12/29 (41%)	7/29 (24%)	6/29 (21%)	23/29 (79%)	4/29 (14%)	3/29 (10%)
Normal neuropathological examination with T2DM*	13/19 (68%)	19/19 (53%)	7/19 (37%)	17/19 (89%)	19/19 (100%)	8/19 (42%)	10/19 (53%)	6/19 (32%)	15/19 (79%)	6/19 (32%)	3/19 (16%)
Alzheimer disease with a low neuropathologic change*	22/28 (79%)	24/28 (86%)	14/28 (50%)	21/28 (75%)	28/28 (100%)	17/28 (61%)	21/28 (75%)	11/28 (39%)	24/28 (86%)	9/28 (32%)	10/28 (36%)
Alzheimer disease with an intermediate neuropathologic change*	22/26 (85)	24/26 (92%)	14/26 (54%)	23/26 (88%)	26/26 (100%)	18/26 (69%)	22/26 (85%)	12/26 (46%)	25/26 (96%)	12/26 (46%)	10/26 (38%)
Alzheimer disease with a high neuropathologic change*	8/8 (100%)	8/8 (100%)	5/8 (63%)	8/8 (100%)	8/8 (100%)	4/8 (50%)	7/8 (88%)	7/8 (88%)	8/8 (100%)	7/8 (88%)	4/8 (50%)

The data are represented as n (%). PD, Parkinson’s disease; DLB, dementia with Lewy bodies; ILBD, incidental Lewy body disease. Tau AT-8, Ser202-Thr205-phosphorylated tau; Tau AT-100, Thr212-Ser214-phosphorylated tau; Tau AT-180, Thr231-phosphorylated tau; Tau AT-270, Thr181-phosphorylated tau; and pTau, phosphorylated tau. *These results had been previously published in Martinez-Valbuena et al. 2019 and have been added in this table to facilitate comparisons between groups

### PrP is primarily expressed in pancreatic β-cells and binds to amylin, α-synuclein, tau and Aβ either in the pancreas or in the *locus coeruleus*

**Fig. 4:**
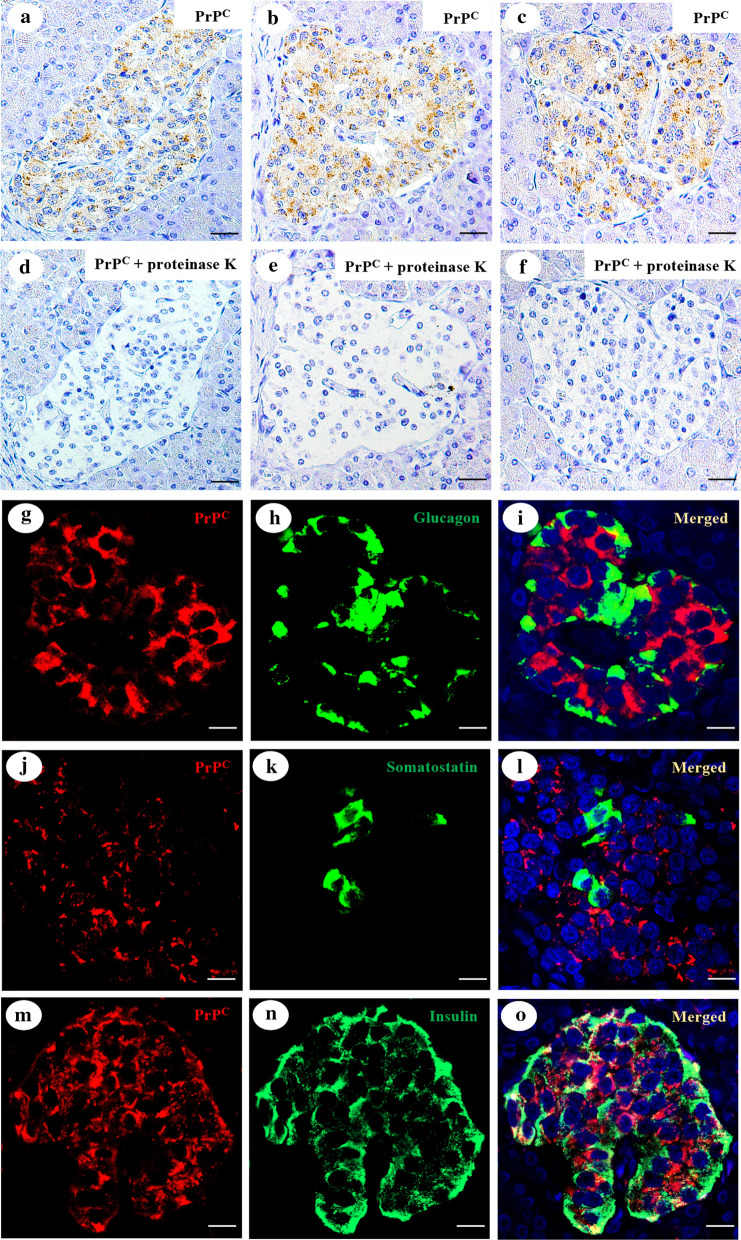
PrP pancreatic expression characterization. Immunohistochemistry for PrP with (**a**–**c**) and without (**d**–**f**) proteinase K digestion in the pancreas from a subject with a normal neuropathological examination and history of T2DM (**a**, **d**), a subject with Parkinson’s disease (**b**, **e**) and with Alzheimer’s disease (**c**, **f**). Dual immunofluorescence for PrP^c^ and glucagon (**g**–**i**), somatostatin (**j**–**l**) or insulin (**m**–**o**) to assess the cellular localization of PrP in the pancreas from a 73-year-old female with Parkinson’s disease: 40 × magnification; scale bar = 50 µm

In addition to the evaluation of PrP pancreatic immunoreactivity, we performed a proteinase K treatment (Fig. 4a–f) on one section from each of the subjects included in the study to determine if the pancreatic PrP was cellular PrP (PrP^c^) or misfolded PrP (PrP^Sc^). We found that pancreatic PrP was proteinase K sensitive in all cases, regardless of the neuropathologic diagnosis, indicating that pancreatic PrP was not misfolded. Furthermore, to determine which endocrine pancreatic cell type expressed PrP, we performed double immunofluorescence staining for PrP and each of the three major pancreatic hormones: glucagon (produced by α cells, Fig. 4g–i), somatostatin (produced by δ cells, Fig. 4j–l) and insulin (produced by β cells, Fig. 4m–o), finding that PrP expression was primarily detected in the latter ones.

**Fig. 5:**
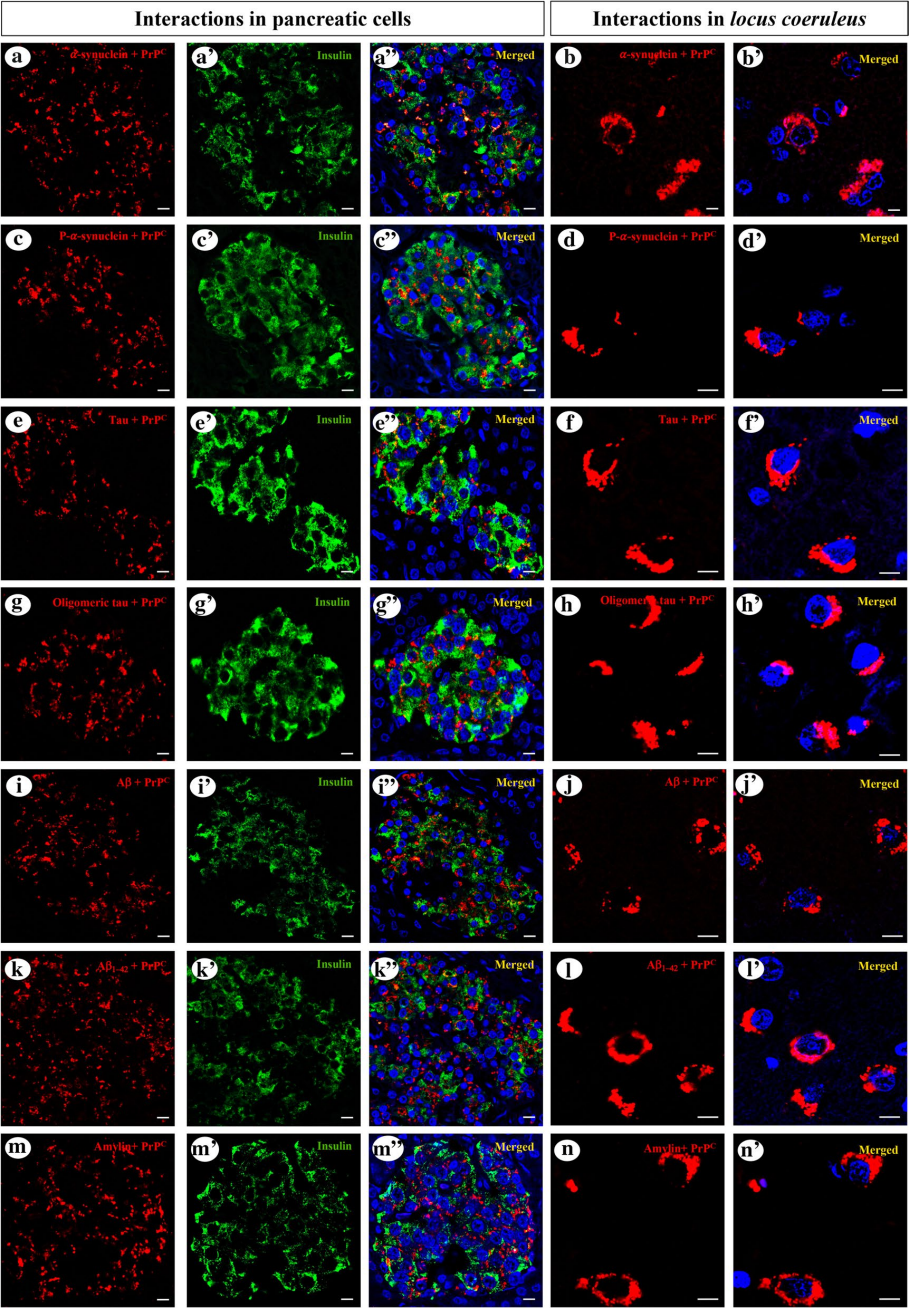
In situ proximity ligation assays (PLA) on pancreatic β cells and the *locus coeruleus*. PLA for PrP and α-synuclein (**a**–**a**ʺ), serine 129-phosphorylated α-synuclein (**c**–**c**ʺ), tau (**e**–**e**ʺ), oligomeric tau (**g**–**g**ʺ), Aβ (**i**–**i**ʺ), Aβ_1-42_ (**k**–**k**ʺ) or amylin (**m**–**m**ʺ) with insulin immunofluorescence showed an interaction between these proteins in pancreatic β cells from a 73-year-old female with Parkinson’s disease (**a**ʺ, **c**ʺ) and from a 70-year-old male with Alzheimer’s disease (**e**ʺ, **g**ʺ, **i**ʺ, **k**ʺ, **m**ʺ). Interactions between PrP and α-synuclein (**b**ʹ, **d**ʹ), tau (**f**ʹ, **h**ʹ), Aβ (**j**ʹ, **l**ʹ) or amylin (**n**ʹ) were found in the *locus coeruleus* of the same subjects: DAPI nuclear counterstaining was used, 40 × magnification; scale bar = 50 µm

To assess whether in addition to their co-expression in pancreatic β cells, there was a direct interaction between PrP and amylin, tau, α-synuclein, Aβ or their pathological conformers (phosphorylated α-synuclein, oligomeric tau and the Aβ_1-42_ peptide) in pancreatic and brain tissue we performed PLA assays in 3 subjects per group. To ensure the specificity of the PrP PLA, we performed an assay between a protein constitutively expressed in pancreatic β cells (CgA) and PrP. While all the PLA assays performed with CgA were negative (Additional file 1: Fig. S3), both total and pathological forms of tau, α-synuclein and Aβ, together with amylin, appeared to interact with PrP in pancreatic β-cells and in the *locus coeruleus* in all subjects with protein inclusions, regardless the neuropathological diagnosis (Fig. 5a–nʺ).

## Discussion

In this retrospective autopsy-based study, we show, for the first time, the existence of cytoplasmic tau and Aβ protein deposits in pancreatic β-cells of subjects with synucleinopathies, as well as the existence of cytoplasmic α-synuclein inclusions in subjects with AD. Furthermore, we present histological evidence of PrP interactions with α-synuclein, Tau, Aβ and amylin in both pancreatic β-cells and in the *locus coeruleus*.

The link between neurodegenerative diseases and T2DM has long been recognized in epidemiological studies [38, 49], but the mechanisms for this comorbidity are not yet fully elucidated. T2DM is a metabolic disease characterized by chronic insulin resistance and pancreatic β-cell dysfunction, leading to impaired insulin release and hyperglycemia [44]. The cause of this β-cell loss is not completely understood, although several studies have implicated amylin aggregation in this process [12]. Amylin is a small and highly amyloidogenic protein secreted from β-cells alongside insulin, and it regulates gastric emptying and glucose homeostasis [44]. However, in addition to amylin misfolding and deposition, a sizeable body of evidence has shown that other amyloidogenic proteins may have important roles in the pathogenesis of this chronic disease [10, 63, 69, 70]. Therefore, in this study, we conducted a semiquantitative analysis to assess the immunoreactivity of four amyloidogenic proteins expressed in the pancreas: amylin, α-synuclein, tau and PrP. The wider immunoreactivity found in pancreatic tissue was of amylin, and as expected, our results showed an increase in amylin immunoreactivity in T2DM subjects compared to subjects with neurodegenerative diseases and controls. However, interestingly, we found an increased immunoreactivity of amylin in subjects with neurodegenerative diseases compared to controls. When α-synuclein immunoreactivity was assessed, we found that subjects with a synucleinopathy showed the highest α-synuclein pancreatic immunoreactivity compared to controls, subjects with a normal neuropathological examination and a history of T2DM and subjects with AD. Likewise, when tau immunoreactivity was assessed, subjects with AD had the highest immunoreactivity among all the subjects included. These results show that in the pancreatic β-cells of subjects with T2DM or neurodegenerative diseases, there is an overall increase in the immunoreactivity of amyloidogenic proteins compared to control subjects and that a specific overexpression of the disease-associated protein over the rest is found in each group of patients. This increase in the pancreatic immunoreactivity of amyloidogenic proteins could have implications in the pathophysiology of T2DM and in the development of insulin resistance by patients with AD or a synucleinopathy, due to the fact that in some patients the threshold for a clinical impairment could be reached by a sufficient amount of a single misfolded protein (i.e. amylin) in pancreatic β-cells; however, in other cases, it could be reached by the combined and additive presence of various misfolded proteins that in isolation are not sufficient to cause clinical symptoms, as has been proposed for some neurodegenerative conditions [31, 32]. Although the present study cannot either unveil whether these upregulations were transcriptionally regulated or instead resulted from a decrease in protein clearance, and the underlying mechanisms and the consequences of this protein-specific increment in each group of patients, some of the functional consequences of an increase in the expression of these proteins in pancreatic β cells have already been described. For example, enhanced α-synuclein expression in the pancreatic β-cells of mice impairs glucose-stimulated insulin secretion, and it negatively influences microtubule polymerization and trafficking, mechanisms implicated in both insulin secretion and autophagy [62]. Likewise, increased tau levels in β-cells of T2DM patients have been described [40], and this enhanced tau expression might influence the rate of insulin gene transcription and regulate its trafficking [70].

As our group previously described the presence of pancreatic α-synuclein deposits in subjects with synucleinopathies, as well as deposits of tau and Aβ in subjects with AD [39, 40], we also conducted a examination to evaluate whether in addition to an increase in total protein immunoreactivity, we also found mixed pathologies in the pancreatic β-cells of subjects with neurodegenerative diseases. Consequently, we performed a detailed histopathological examination of pancreatic phosphorylated α-synuclein inclusions with two different validated antibodies in AD subjects. We found that approximately 40% of AD subjects with low neuropathologic change and half of the AD subjects with intermediate neuropathologic changes had phosphorylated α-synuclein pancreatic inclusions but that 75% of subjects with AD and high neuropathologic change had these inclusions. Interestingly, the prevalence of phosphorylated α-synuclein pancreatic inclusions is in line with those reported in autopsies of AD subjects, where phosphorylated α-synuclein inclusions ranged from 30 to 70% [2, 32, 54]. Furthermore, in addition to phosphorylation, α-synuclein undergoes a variety of posttranslational modifications, such as truncation and nitration, that may have an important role in its misfolding [26, 27]. Therefore, we expanded the histopathological assessment and examined either truncated or nitrated α-synuclein in the pancreas of all the subjects included in the study. C-terminal-truncated α-synuclein immunoreactivity was present in the pancreas of more than 80% of subjects with T2DM or neurodegenerative diseases, and while nitrated α-synuclein immunoreactivity was only found in 15% of control subjects, it was detected in more than 50% of subjects with PD, DLB or AD with intermediate or high neuropathologic changes, evidence that C-terminal truncation and nitration of α-synuclein play a role in α-synuclein misfolding [71]. To determine whether, in addition to phosphorylation, nitration and cleavage, α-synuclein in the pancreas and brain undergoes similar conformational changes, we performed α-synuclein-PLA in pancreatic tissue. This assay detects α-synuclein oligomeric pathology in neuroanatomical areas ahead of regular immunohistochemistry, providing an extra level of detail and resolution of α-synuclein pathology [55]. When we performed the assay, we found that α-synuclein oligomers were mainly present in the cytoplasm of pancreatic β-cells of subjects with neurodegenerative diseases and, as previously described [62], in subjects with T2DM. Furthermore, we also described thioflavin-positive deposits of α-synuclein in β-cells of AD patients.

On the other hand, we evaluated the presence of Aβ and Tau deposits in patients with synucleinopathies. We found pancreatic deposits of Aβ in more than 25% of these patients. Thereafter, we performed a detailed histopathological assessment of the expression of tau deposits in pancreatic tissue from patients with synucleinopathies using ten different validated antibodies that recognize different conformations and phosphorylation sites. Cytoplasmic expression of Thr181- and Ser262-hyperphosphorylated tau was detected in pancreatic cells from all subjects with synucleinopathies. These two early phosphorylation events seem to be associated with pretangle formation [25], yet other epitopes thought to be phosphorylated at later stages of the disease [25] had a greater presence in pancreatic β-cells of subjects with DLB (e.g., Ser202-Thr205, Thr212-Ser214, Thr231 or Ser422) than in patients with ILBD or control subjects. The same pattern was observed for tau truncated at Asp421, which was mostly detected in subjects with DLB and PD but also in 75% of subjects with ILBD. The presence of truncated forms of tau in pancreatic cells is particularly relevant, and while their role has not been fully elucidated, it has been suggested that truncated protein fragments may represent the initial seeds of tau aggregates [16]. To determine whether the tau in the pancreas suffers conformational changes in addition to phosphorylation and cleavage, we used the MC1 and Alz50 antibodies that recognize abnormal tau conformations [30]. Alz50 immunoreactivity was present in the pancreas of more than 75% of subjects, evidence that it is an early indicator of tau misfolding [25]. Conversely, the MC1 antibody detects advanced conformational changes in tau prior to tau aggregates becoming argyrophilic [30], and while MC1 immunoreactivity was found in 42% of subjects with DLB, such isoforms were detected in more than 85% of subjects with AD with high neuropathologic changes, suggesting that although subjects with synucleinopathies have hyperphosphorylated tau deposits, as happens in the brain, some conformational changes seem to be specific to AD subjects, even in pancreatic β-cells. Furthermore, as evaluated for α-synuclein oligomers, we also examined whether tau oligomers were assembled in pancreatic β-cells, and interestingly, we detected tau oligomers in more than 80% of subjects with synucleinopathies. All these data suggest that in addition to the increase in the protein levels, post-translational modifications of either α-synuclein, Aβ and tau would also play a key role in T2DM pathophysiology and the development of insulin resistance in patients with neurodegenerative diseases.

Finally, we found that in all cases, pancreatic PrP was sensitive to proteinase K and that it was mainly expressed in the cytoplasm of pancreatic β-cells. Pancreatic PrP immunoreactivity was more abundant in subjects with a normal neuropathological examination and a history of T2DM than in controls. However, subjects with synucleinopathies or with AD had wider PrP expression than subjects with T2DM. As the major overexpression was found in subjects with neurodegenerative diseases, who were also the ones who had more misfolded Aβ, tau and α-synuclein, we performed PLA assays to determine whether there was a direct interaction between PrP and amylin, tau, α-synuclein or Aβ in pancreatic tissue in addition to co-expression in pancreatic β-cells, as has been described in brain tissue [17, 24]. Our data showed that in addition to total tau, α-synuclein, amylin and Aβ, PrP also interacted specifically with phosphorylated α-synuclein, oligomeric tau and the Aβ_1-42_ peptide. Both total and pathological forms of tau, α-synuclein and Aβ, together with amylin, appeared to interact with PrP in pancreatic β-cells and in the *locus coeruleus.* Unfortunately, we were not able to provide the mechanistic insights of these interactions, and although other studies [36, 65] had also shown the existence of a binding between PrP and Aβ, tau and α-synuclein,, the functional consequences still remain controversial [5]. This could be caused by the coexistence of both PrP-dependent and PrP-independent neuropathological mechanisms for oligomer neurotoxicity [52, 67], and that it is still unclear whether different protein conformations or strains may all be recognized by PrP [27]. Additional studies detailing the molecular interactions between different strains of amyloidogenic proteins and PrP and the relevance of amylin binding to PrP either in the pancreas or in the brain will provide novel insight needed to fully elucidate the role of PrP as a receptor of these misfolded proteins.

Furthermore, larger studies are needed to correlate the pancreatic and brain pathology and if these pancreatic inclusions are correlated with the onset, duration and severity of T2DM or with the presence or absence of insulin resistance in the subjects with neurodegenerative diseases.

In conclusion, this study shows that along with amylin, α-synuclein, Aβ, PrP and tau found in pancreatic β-cells may contribute together to the complex pathophysiology of T2DM and to the appearance of insulin resistance in non-T2DM AD, PD or DLB patients. Furthermore, we show that the same mixed pathologies that are observed in the brains of these patients with neurodegenerative diseases are also present outside the nervous system. Finally, we provide histological evidence of an interaction between PrP and Aβ, α-synuclein, amylin or tau in the pancreas and *locus coeruleus*. Further prospective studies will be necessary to validate these findings, and additional work should be performed to ascertain the relevance of amyloidogenic protein inclusions in T2DM pathophysiology. Furthermore, a deeper understanding of the role of PrP as a receptor of amyloidogenic proteins either in the pancreas or in the brain will also shed more light on the common pathological pathways shared by neurodegenerative diseases and T2DM, benefiting the exploration of common therapeutic strategies to prevent or treat these devastating amyloid diseases.

## Supplementary Information

**Additional file 1.** Supplementary figures and tables.
