# Supplementary material for: Mixed pathologies in pancreatic β cells from subjects with neurodegenerative diseases and their interaction with prion protein
Source: Acta Neuropathol Commun. 2021 Apr 8;9:64. doi: 10.1186/s40478-021-01171-0 (PMC8028740; doi:10.1186/s40478-021-01171-0)

**Supplementary material**

**Tables**

**Table S1.** Demographic, clinical and neuropathological diagnosis of the subjects included in this study. (T2DM: type two diabetes mellitus; F: female; M: male; PMD: post-mortem delay).

| **Subject ID** | **Age (years)** | **Sex** | **T2DM** | **Anti-diabetic drug** | **PMD**  **(hours)** | **Cause of death** | **Neuropathological diagnosis** |
| --- | --- | --- | --- | --- | --- | --- | --- |
| 1 | 70 | F | No | No | 3 | Heart attack | Parkinson's disease (Braak 4) |
| 2 | 74 | M | No | No | 3,2 | Massive hemorrhage | Alzheimer disease neuropathologic change intermediate (A3, B2, C2) |
| 3 | 86 | M | No | No | 8 | Heart attack | Alzheimer disease neuropathologic change intermediate (A2, B3, C2) |
| 4 | 82 | F | No | No | 9 | Pneumonia | Parkinson's disease (Braak 3) |
| 5 | 82 | M | No | No | 8 | Pulmonary thromboembolism | Dementia with Lewy bodies (brainstem-predominant) |
| 6 | 95 | F | No | No | 4 | Heart attack | Alzheimer disease neuropathologic change high (A3, B3, C2) |
| 7 | 79 | M | Yes | Insulin | 6,3 | Heart attack | Normal neuropathological examination |
| 8 | 81 | F | No | No | 7 | Breast carcinoma | Dementia with Lewy bodies (brainstem-predominant) |
| 9 | 82 | M | No | No | 5 | Pneumonia | Parkinson's disease (Braak 4) |
| 10 | 78 | F | Yes | Glimepiride | 4 | Pneumonia | Normal neuropathological examination |
| 11 | 78 | F | No | No | 4 | Septic shock | Alzheimer disease neuropathologic change intermediate (A2, B2, C2) |
| 12 | 75 | F | No | No | 9,5 | Pyelonephritis | Normal neuropathological examination |
| 13 | 92 | F | No | No | 3 | Digestive bleeding | Alzheimer disease neuropathologic change high (A3, B3, C2) |
| 14 | 90 | F | No | No | 5,5 | Massive hemorrhage | Alzheimer disease neuropathologic change intermediate (A3, B2, C3) |
| 15 | 73 | M | No | No | 4,5 | Pneumonia | Dementia with Lewy bodies (brainstem-predominant) |
| 16 | 80 | F | No | No | 5 | Hepatic carcinoma | Parkinson's disease (Braak 4) |
| 17 | 77 | F | No | No | 5, 2 | Heart failure | Alzheimer disease neuropathologic change intermediate (A2, B3, C2) |
| 18 | 82 | M | No | No | 3,4 | Heart failure | Alzheimer disease neuropathologic change high (A3, B3, C3) |
| 19 | 73 | M | No | No | 4,3 | Heart failure | Normal neuropathological examination |
| 20 | 68 | M | No | No | 3,5 | Hypovolemic shock | Normal neuropathological examination |
| 21 | 79 | F | No | No | 8,3 | Necrotizing enterocolitis | Normal neuropathological examination |
| 22 | 72 | F | No | No | 6,5 | Heart attack | Alzheimer disease neuropathologic change intermediate (A2, B2, C3) |
| 23 | 79 | M | No | No | 5,5 | Pneumonia | Normal neuropathological examination |
| 24 | 76 | M | No | No | 3,2 | Heart attack | Normal neuropathological examination |
| 25 | 74 | M | No | No | 6,5 | Digestive bleeding | Alzheimer disease neuropathologic change low (A1, B1, C1) |
| 26 | 81 | M | No | No | 3,4 | Pneumonia | Normal neuropathological examination |
| 27 | 67 | M | Yes | Glicazide | 9 | Heart attack | Normal neuropathological examination |
| 28 | 73 | M | No | No | 9 | Lung carcinoma | Alzheimer disease neuropathologic change low (A1, B2, C1) |
| 29 | 77 | F | No | No | 12 | Heart attack | Alzheimer disease neuropathologic change low (A2, B1, C1) |
| 30 | 80 | F | No | No | 7 | Septic shock | Normal neuropathological examination |
| 31 | 79 | F | No | No | 6 | Pulmonary thromboembolism | Alzheimer disease neuropathologic change high (A3, B3, C3) |
| 32 | 64 | F | Yes | Insulin | 4,3 | Lung carcinoma | Normal neuropathological examination |
| 33 | 77 | M | No | No | 5 | Heart attack | Alzheimer disease neuropathologic change intermediate (A1, B2, C2) |
| 34 | 64 | F | No | No | 6,2 | Colon carcinoma | Normal neuropathological examination |
| 35 | 91 | M | No | No | 5 | Pneumonia | Alzheimer disease neuropathologic change high (A3, B3, C3) |
| 36 | 75 | M | No | No | 3,5 | Pneumonia | Alzheimer disease neuropathologic change low (A1, B2, C1) |
| 37 | 82 | F | No | No | 4 | Pulmonary thromboembolism | Alzheimer disease neuropathologic change intermediate (A2, B2, C1) |
| 38 | 69 | F | No | No | 8,3 | Heart attack | Alzheimer disease neuropathologic change low (A1, B2, C1) |
| 39 | 71 | M | No | No | 12 | Heart failure | Alzheimer disease neuropathologic change low (A1, B1, C1) |
| 40 | 68 | F | Yes | Insulin | 9 | Heart attack | Normal neuropathological examination |
| 41 | 79 | F | No | No | 4,5 | Septic shock | Alzheimer disease neuropathologic change low (A1, B1, C0) |
| 42 | 88 | F | No | No | 3,5 | Pneumonia | Dementia with Lewy bodies (brainstem-predominant) |
| 43 | 73 | M | No | No | 7 | Heart failure | Normal neuropathological examination |
| 44 | 84 | F | No | No | 14 | Heart attack | Alzheimer disease neuropathologic change low (A1, B2, C1) |
| 45 | 79 | F | No | No | 5 | Pulmonary fibrosis | Parkinson's disease (Braak 4) |
| 46 | 75 | M | No | No | 4 | Colon carcinoma | Alzheimer disease neuropathologic change low (A1, B1, C1) |
| 47 | 87 | F | No | No | 4 ,5 | Pulmonary thromboemolism | Alzheimer disease neuropathologic change intermediate (A2, B2, C2) |
| 48 | 68 | M | No | No | 2 | Pneumonia | Normal neuropathological examination |
| 49 | 77 | M | Yes | Glimepiride | 6,2 | Heart attack | Normal neuropathological examination |
| 50 | 81 | M | No | No | 7,5 | Aortic stenosis | Incidental Lewy bodies disease (Braak 1) |
| 51 | 61 | M | Yes | Glibenclamide | 8 | Septic shock | Normal neuropathological examination |
| 52 | 66 | M | No | No | 4,3 | Heart failure | Normal neuropathological examination |
| 53 | 87 | F | No | No | 6 | Heart attack | Alzheimer disease neuropathologic change intermediate (A2, B3, C1) |
| 54 | 72 | M | No | No | 8 | Pneumonia | Normal neuropathological examination |
| 55 | 76 | F | No | No | 6 | Septic shock | Alzheimer disease neuropathologic change intermediate (A3, B2, C2) |
| 56 | 77 | F | No | No | 4 | Pulmonary thromboemolism | Alzheimer disease neuropathologic change intermediate (A2, B3, C2) |
| 57 | 80 | M | No | No | 5,2 | Heart failure | Alzheimer disease neuropathologic change intermediate (A2, B3, C1) |
| 58 | 79 | F | No | No | 3,5 | Multiple organ dysfunction syndrome | Alzheimer disease neuropathologic change intermediate (A2, B2, C2) |
| 59 | 61 | M | No | No | 4 | Heart attack | Normal neuropathological examination |
| 60 | 88 | F | No | No | 5 | Heart attack | Alzheimer disease neuropathologic change low (A1, B2, C1) |
| 61 | 74 | F | No | No | 4 | Septic shock | Alzheimer disease neuropathologic change intermediate (A2, B3, C2) |
| 62 | 69 | M | No | No | 4 | Esophageal carcinoma | Alzheimer disease neuropathologic change low (A1, B1, C1) |
| 63 | 71 | M | Yes | Glipizide | 6,2 | Post-surgical cardiogenic stroke | Normal neuropathological examination |
| 64 | 60 | M | No | No | 3,5 | Heart failure | Alzheimer disease neuropathologic change intermediate (A2, B2, C1) |
| 65 | 82 | F | No | No | 10 | Pneumonia | Alzheimer disease neuropathologic change intermediate (A2, B2, C1) |
| 66 | 72 | M | Yes | Acarbose | 7,5 | Pneumonia | Normal neuropathological examination |
| 67 | 73 | F | No | No | 4,2 | Hepatic carcinoma | Alzheimer disease neuropathologic change low (A1, B1, C1) |
| 68 | 81 | F | No | No | 6,2 | Septic shock | Alzheimer disease neuropathologic change low (A1, B3, C1) |
| 69 | 71 | M | Yes | Glimepiride | 4,5 | Heart attack | Normal neuropathological examination |
| 70 | 81 | M | No | No | 2,5 | Pneumonia | Alzheimer disease neuropathologic change low (A1, B2, C1) |
| 71 | 73 | F | No | No | 6 | Heart attack | Normal neuropathological examination |
| 72 | 74 | M | No | No | 4 | Lung carcinoma | Alzheimer disease neuropathologic change low (A1, B1, C1) |
| 73 | 81 | M | No | No | 5, 2 | Pneumonia | Alzheimer disease neuropathologic change low (A2, B1, C1) |
| 74 | 72 | M | No | No | 3,5 | Heart failure | Alzheimer disease neuropathologic change intermediate (A2, B3, C2) |
| 75 | 79 | M | No | No | 6 | Pneumonia | Alzheimer disease neuropathologic change intermediate (A1, B2, C2) |
| 76 | 64 | M | No | No | 7 | Pneumonia | Normal neuropathological examination |
| 77 | 70 | M | No | No | 3 | Heart attack | Normal neuropathological examination |
| 78 | 80 | F | No | No | 6,5 | Pulmonary thromboemolism | Normal neuropathological examination |
| 79 | 66 | M | No | No | 4 | Pyelonephritis | Normal neuropathological examination |
| 80 | 72 | M | No | No | 3 | Prostatic carcinoma | Alzheimer disease neuropathologic change low (A1, B1, C1) |
| 81 | 65 | M | No | No | 11 | Renal disease | Normal neuropathological examination |
| 82 | 61 | M | No | No | 10 | Heart attack | Normal neuropathological examination |
| 83 | 85 | F | No | No | 7 | Pneumonia | Alzheimer disease neuropathologic change low (A1, B2, C1) |
| 84 | 74 | F | Yes | Acarbose | 3,2 | Heart failure | Normal neuropathological examination |
| 85 | 76 | M | No | No | 6,2 | Pneumonia | Parkinson's disease (Braak 3) |
| 86 | 88 | M | No | No | 4,5 | Heart attack | Alzheimer disease neuropathologic change intermediate (A2, B2, C2) |
| 87 | 70 | M | No | No | 3,4 | Heart attack | Normal neuropathological examination |
| 88 | 60 | F | Yes | Acarbose | 8 | Septic shock | Normal neuropathological examination |
| 89 | 60 | F | Yes | Glimepiride | 9 | Pulmonary thromboembolism | Normal neuropathological examination |
| 90 | 75 | F | No | No | 4,5 | Pulmonary congestion | Dementia with Lewy bodies (brainstem-predominant) |
| 91 | 76 | M | No | No | 4,5 | Heart attack | Alzheimer disease neuropathologic change low (A1, B2, C1) |
| 92 | 84 | M | No | No | 5,2 | Pneumonia | Alzheimer disease neuropathologic change low (A1, B2, C1) |
| 93 | 86 | M | No | No | 7 | Heart failure | Dementia with Lewy bodies (brainstem-predominant) |
| 94 | 63 | F | No | No | 3,4 | Heart attack | Normal neuropathological examination |
| 95 | 70 | F | No | No | 6,3 | Valvular cardiopathy | Incidental Lewy bodies disease (Braak 1) |
| 96 | 75 | F | No | No | 7,5 | Heart attack | Alzheimer disease neuropathologic change intermediate (A2, B3, C2) |
| 97 | 61 | M | Yes | Insulin | 4 | Neuloleptic malignant syndrome | Normal neuropathological examination |
| 98 | 84 | M | No | No | 2,5 | Heart failure | Dementia with Lewy bodies (brainstem-predominant) |
| 99 | 72 | M | No | No | 5, 2 | Pulmonary thromboembolism | Dementia with Lewy bodies (brainstem-predominant) |
| 100 | 67 | M | No | No | 3,2 | Post surgical septic shock | Incidental Lewy bodies disease (Braak 1) |
| 101 | 78 | M | No | No | 4 ,5 | Septic shock | Alzheimer disease neuropathologic change intermediate (A2, B3, C3) |
| 102 | 73 | F | Yes | Yes | 5, 2 | Heart failure | Normal neuropathological examination |
| 103 | 75 | M | No | No | 3,4 | Heart attack | Parkinson's disease (Braak 3) |
| 104 | 63 | M | Yes | No | 3,2 | Pneumonia | Normal neuropathological examination |
| 105 | 64 | F | No | No | 7 | Digestive bleeding | Alzheimer disease neuropathologic change high (A3, B3, C3) |
| 106 | 66 | M | No | No | 6 | Renal oncocitosis | Normal neuropathological examination |
| 107 | 62 | M | Yes | Insulin | 9 | Heart attack | Normal neuropathological examination |
| 108 | 76 | M | No | No | 5, 2 | Lung carcinoma | Incidental Lewy bodies disease (Braak 1) |
| 109 | 72 | M | No | No | 10 | Digestive bleeding | Normal neuropathological examination |
| 110 | 95 | F | No | No | 6 | Post surgical cardiogenic stroke | Alzheimer disease neuropathologic change low (A1, B2, C1) |
| 111 | 79 | M | No | No | 11 | Pulmonary thromboembolism | Alzheimer disease neuropathologic change low (A1, B3, C1) |
| 112 | 81 | F | Yes | Insulin | 2,5 | Heart failure | Normal neuropathological examination |
| 113 | 84 | M | No | No | 8,5 | Bowel obstruction | Dementia with Lewy bodies (brainstem-predominant) |
| 114 | 72 | F | No | No | 4 | Heart attack | Alzheimer disease neuropathologic change intermediate (A1, B2, C1) |
| 115 | 84 | F | No | No | 6,2 | Multiple organ dysfunction syndrome | Alzheimer disease neuropathologic change high (A3, B3, C3) |
| 116 | 73 | F | No | No | 6,5 | Heart attack | Alzheimer disease neuropathologic change low (A1, B2, C1) |
| 117 | 70 | M | No | No | 3 | Heart attack | Normal neuropathological examination |
| 118 | 73 | M | Yes | Repaglinide | 6,5 | Aspiration pneumonia | Normal neuropathological examination |
| 119 | 67 | M | No | No | 4 | Pulmonary thromboembolism | Alzheimer disease neuropathologic change low (A1, B2, C1) |
| 120 | 77 | M | No | No | 5,5 | Post surgical cardiogenic stroke | Alzheimer disease neuropathologic change intermediate (A2, B2, C2) |
| 121 | 76 | F | No | No | 4 | Lung carcinoma | Alzheimer disease neuropathologic change low (A1, B1, C1) |
| 122 | 76 | F | No | No | 6,2 | Heart attack | Alzheimer disease neuropathologic change low (A1, B1, C1) |
| 123 | 75 | M | No | No | 3,4 | Massive hemorrhage | Alzheimer disease neuropathologic change high (A3, B3, C3) |
| 124 | 78 | M | No | No | 8,3 | Aspiration pneumonia | Dementia with Lewy bodies (brainstem-predominant) |
| 125 | 62 | M | No | No | 5,4 | Aortic dissection | Parkinson's disease (Braak 3) |
| 126 | 71 | F | No | No | 2,5 | Septic shock | Alzheimer disease neuropathologic change low (A1, B1, C0) |
| 127 | 63 | F | No | No | 11 | Heart attack | Normal neuropathological examination |
| 128 | 84 | M | No | No | 9 | Heart attack | Parkinson's disease (Braak 4) |
| 129 | 76 | F | No | No | 11 | Septic shock | Alzheimer disease neuropathologic change low (A1, B2, C1) |
| 130 | 80 | F | No | No | 4,2 | Aspiration pneumonia | Alzheimer disease neuropathologic change intermediate (A2, B2, C2) |
| 131 | 90 | M | No | No | 7 | Cardiac thrombosis | Dementia with Lewy bodies (limbic) |
| 132 | 66 | M | No | No | 9,5 | Bladder carcinoma | Incidental Lewy bodies disease (Braak 2) |
| 133 | 73 | M | No | No | 6 | Mitocondrial cardiomyopathy | Incidental Lewy bodies disease (Braak 2) |
| 134 | 76 | M | No | No | 8,3 | Heart attack | Incidental Lewy bodies disease (Braak 2) |
| 135 | 61 | M | No | No | 7 | Lung carcinoma | Incidental Lewy bodies disease (Braak 2) |
| 136 | 77 | M | No | No | 6,5 | Renal oncocitosis | Alzheimer disease neuropathologic change intermediate (A2, B2, C2) |
| 137 | 82 | F | No | No | 8 | Cirrhosis | Parkinson's disease (Braak 3-4) |
| 138 | 88 | M | No | No | 8 | Pulmonary thromboembolism | Dementia with Lewy bodies (brainstem-predominant) |

**Table S2.** Neuropathological characteristics of the subjects included in this study.

| **Subject ID** | **Braak and Braak NFT stage** | **Thal phase for Aβ plaques** | **CERAD neuritic plaque score** | **Braak stage for Lewy pathology** |
| --- | --- | --- | --- | --- |
| 1 | 0 | 0 | 0 | 4 |
| 2 | IV | 4 | 2 | 2 |
| 3 | III | 4 | 2 | 1 |
| 4 | I | 0 | 0 | 3 |
| 5 | I | 1 | 1 | 5 |
| 6 | V | 5 | 2 | 2 |
| 7 | 0 | 0 | 0 | 0 |
| 8 | 0 | 0 | 0 | 6 |
| 9 | I | 1 | 0 | 4 |
| 10 | I | 0 | 0 | 0 |
| 11 | III | 3 | 2 | 0 |
| 12 | I | 0 | 0 | 0 |
| 13 | IV | 5 | 2 | 0 |
| 14 | III | 5 | 3 | 0 |
| 15 | II | 2 | 1 | 6 |
| 16 | 0 | 0 | 0 | 4 |
| 17 | IV | 3 | 2 | 2 |
| 18 | VI | 5 | 3 | 2 |
| 19 | I | 0 | 0 | 0 |
| 20 | 0 | 0 | 0 | 0 |
| 21 | I | 0 | 0 | 0 |
| 22 | IV | 3 | 3 | 2 |
| 23 | 0 | 0 | 0 | 0 |
| 24 | I | 0 | 0 | 0 |
| 25 | II | 1 | 1 | 0 |
| 26 | 0 | 0 | 0 | 0 |
| 27 | 0 | 0 | 0 | 0 |
| 28 | III | 1 | 1 | 0 |
| 29 | II | 3 | 1 | 0 |
| 30 | I | 0 | 0 | 0 |
| 31 | V | 5 | 3 | 1 |
| 32 | 0 | 0 | 0 | 0 |
| 33 | IV | 1 | 2 | 1 |
| 34 | 0 | 0 | 0 | 0 |
| 35 | V | 5 | 3 | 3 |
| 36 | IV | 2 | 1 | 0 |
| 37 | IV | 3 | 1 | 0 |
| 38 | III | 2 | 1 | 1 |
| 39 | II | 1 | 1 | 1 |
| 40 | 0 | 0 | 0 | 0 |
| 41 | II | 1 | 0 | 2 |
| 42 | II | 2 | 2 | 6 |
| 43 | I | 0 | 0 | 0 |
| 44 | III | 1 | 1 | 0 |
| 45 | 0 | 0 | 0 | 4 |
| 46 | II | 2 | 1 | 0 |
| 47 | III | 3 | 2 | 0 |
| 48 | 0 | 0 | 0 | 0 |
| 49 | 0 | 0 | 0 | 0 |
| 50 | 0 | 1 | 0 | 1 |
| 51 | 0 | 0 | 0 | 0 |
| 52 | 0 | 0 | 0 | 0 |
| 53 | V | 3 | 1 | 2 |
| 54 | I | 0 | 0 | 0 |
| 55 | IV | 4 | 2 | 2 |
| 56 | V | 3 | 2 | 0 |
| 57 | V | 3 | 1 | 1 |
| 58 | IV | 3 | 2 | 0 |
| 59 | 0 | 0 | 0 | 0 |
| 60 | III | 1 | 1 | 0 |
| 61 | V | 4 | 2 | 0 |
| 62 | II | 1 | 1 | 0 |
| 63 | 0 | 0 | 0 | 0 |
| 64 | III | 3 | 1 | 1 |
| 65 | III | 3 | 1 | 0 |
| 66 | I | 0 | 0 | 0 |
| 67 | I | 1 | 1 | 1 |
| 68 | V | 2 | 1 | 1 |
| 69 | 0 | 0 | 0 | 0 |
| 70 | III | 1 | 1 | 0 |
| 71 | 0 | 0 | 0 | 0 |
| 72 | II | 1 | 1 | 0 |
| 73 | II | 3 | 1 | 1 |
| 74 | V | 3 | 2 | 0 |
| 75 | IV | 2 | 2 | 0 |
| 76 | 0 | 0 | 0 | 0 |
| 77 | 0 | 0 | 0 | 0 |
| 78 | I | 0 | 0 | 0 |
| 79 | 0 | 0 | 0 | 0 |
| 80 | I | 1 | 1 | 0 |
| 81 | 0 | 0 | 0 | 0 |
| 82 | 0 | 0 | 0 | 0 |
| 83 | III | 2 | 1 | 0 |
| 84 | I | 0 | 0 | 0 |
| 85 | II | 2 | 2 | 3 |
| 86 | III | 3 | 2 | 2 |
| 87 | 0 | 0 | 0 | 0 |
| 88 | 0 | 0 | 0 | 0 |
| 89 | 0 | 0 | 0 | 0 |
| 90 | I | 2 | 1 | 6 |
| 91 | III | 1 | 1 | 1 |
| 92 | III | 2 | 1 | 2 |
| 93 | III | 3 | 2 | 6 |
| 94 | 0 | 0 | 0 | 0 |
| 95 | I | 1 | 0 | 1 |
| 96 | IV | 3 | 2 | 2 |
| 97 | 0 | 0 | 0 | 0 |
| 98 | II | 2 | 2 | 6 |
| 99 | I | 0 | 0 | 6 |
| 100 | 0 | 0 | 0 | 1 |
| 101 | V | 3 | 3 | 1 |
| 102 | 0 | 0 | 0 | 0 |
| 103 | 0 | 2 | 1 | 3 |
| 104 | 0 | 0 | 0 | 0 |
| 105 | VI | 5 | 3 | 2 |
| 106 | 0 | 0 | 0 | 0 |
| 107 | 0 | 0 | 0 | 0 |
| 108 | 0 | 0 | 0 | 1 |
| 109 | 0 | 0 | 0 | 0 |
| 110 | IV | 2 | 1 | 0 |
| 111 | V | 1 | 1 | 1 |
| 112 | I | 0 | 0 | 0 |
| 113 | III | 3 | 3 | 6 |
| 114 | II | 2 | 1 | 0 |
| 115 | V | 4 | 3 | 1 |
| 116 | II | 1 | 1 | 0 |
| 117 | I | 0 | 0 | 0 |
| 118 | 0 | 0 | 0 | 0 |
| 119 | III | 1 | 1 | 0 |
| 120 | IV | 3 | 2 | 0 |
| 121 | II | 2 | 1 | 0 |
| 122 | II | 2 | 1 | 1 |
| 123 | VI | 4 | 3 | 0 |
| 124 | IV | 4 | 2 | 6 |
| 125 | I | 1 | 0 | 3 |
| 126 | I | 1 | 0 | 1 |
| 127 | 0 | 0 | 0 | 0 |
| 128 | II | 2 | 1 | 4 |
| 129 | III | 2 | 1 | 0 |
| 130 | IV | 3 | 2 | 2 |
| 131 | 0 | 0 | 0 | 5 |
| 132 | II | 2 | 1 | 2 |
| 133 | I | 1 | 0 | 2 |
| 134 | I | 0 | 0 | 2 |
| 135 | 0 | 1 | 0 | 2 |
| 136 | IV | 3 | 2 | 0 |
| 137 | II | 2 | 2 | 3 |
| 138 | I | 0 | 0 | 6 |

**Table S3.** Scores obtained by each subject in the semi-quantitative quantification of phosphorylated α-synuclein (Pα-syn), C-terminal truncated α-synuclein and nitrated α-synuclein immunoreactivity pattern observed in pancreatic β-cells. *These scores had been previously published in *Martinez-Valbuena et al 2018* and have been added for comparative purposes.

| **Subject ID** | **Neuropathological diagnosis** | **Scores** | | |
| --- | --- | --- | --- | --- |
|  |  | **Pα-Syn** | **Truncated α-Syn** | **Nitrated α-Syn** |
| 1 | Parkinson's disease (Braak 4) | 3* | 2 | 1 |
| 2 | Alzheimer disease neuropathologic change intermediate (A3, B2, C2) | 3 | 3 | 1 |
| 3 | Alzheimer disease neuropathologic change intermediate (A2, B3, C2) | 2 | 3 | 1 |
| 4 | Parkinson's disease (Braak 3) | 2* | 3 | 1 |
| 5 | Dementia with Lewy bodies (brainstem-predominant) | 1* | 2 | 0 |
| 6 | Alzheimer disease neuropathologic change high (A3, B3, C2) | 2 | 3 | 1 |
| 7 | Normal neuropathological examination with T2DM | 2* | 2 | 0 |
| 8 | Dementia with Lewy bodies (brainstem-predominant) | 2* | 3 | 0 |
| 9 | Parkinson's disease (Braak 4) | 2* | 3 | 1 |
| 10 | Normal neuropathological examination with T2DM | 0* | 2 | 0 |
| 11 | Alzheimer disease neuropathologic change intermediate (A2, B2, C2) | 0 | 2 | 0 |
| 12 | Normal neuropathological examination | 1* | 2 | 0 |
| 13 | Alzheimer disease neuropathologic change high (A3, B3, C2) | 1 | 3 | 0 |
| 14 | Alzheimer disease neuropathologic change intermediate (A3, B2, C3) | 1 | 2 | 0 |
| 15 | Dementia with Lewy bodies (brainstem-predominant) | 1* | 2 | 1 |
| 16 | Parkinson's disease (Braak 4) | 3* | 3 | 0 |
| 17 | Alzheimer disease neuropathologic change intermediate (A2, B3, C2) | 3 | 3 | 1 |
| 18 | Alzheimer disease neuropathologic change high (A3, B3, C3) | 1 | 3 | 1 |
| 19 | Normal neuropathological examination | 2* | 3 | 0 |
| 20 | Normal neuropathological examination | 0* | 0 | 0 |
| 21 | Normal neuropathological examination | 2* | 3 | 1 |
| 22 | Alzheimer disease neuropathologic change intermediate (A2, B2, C3) | 3 | 3 | 1 |
| 23 | Normal neuropathological examination | 0* | 0 | 0 |
| 24 | Normal neuropathological examination | 0* | 0 | 0 |
| 25 | Alzheimer disease neuropathologic change low (A1, B1, C1) | 0 | 2 | 0 |
| 26 | Normal neuropathological examination | 0* | 0 | 0 |
| 27 | Normal neuropathological examination with T2DM | 0* | 0 | 0 |
| 28 | Alzheimer disease neuropathologic change low (A1, B2, C1) | 0 | 2 | 0 |
| 29 | Alzheimer disease neuropathologic change low (A2, B1, C1) | 0 | 2 | 0 |
| 30 | Normal neuropathological examination | 0* | 2 | 0 |
| 31 | Alzheimer disease neuropathologic change high (A3, B3, C3) | 1 | 1 | 1 |
| 32 | Normal neuropathological examination with T2DM | 1* | 3 | 2 |
| 33 | Alzheimer disease neuropathologic change intermediate (A1, B2, C2) | 1 | 3 | 1 |
| 34 | Normal neuropathological examination | 0* | 3 | 0 |
| 35 | Alzheimer disease neuropathologic change high (A3, B3, C3) | 0 | 2 | 1 |
| 36 | Alzheimer disease neuropathologic change low (A1, B2, C1) | 0 | 2 | 0 |
| 37 | Alzheimer disease neuropathologic change intermediate (A2, B2, C1) | 0 | 2 | 0 |
| 38 | Alzheimer disease neuropathologic change low (A1, B2, C1) | 1 | 3 | 0 |
| 39 | Alzheimer disease neuropathologic change low (A1, B1, C1) | 1 | 3 | 0 |
| 40 | Normal neuropathological examination with T2DM | 1* | 2 | 0 |
| 41 | Alzheimer disease neuropathologic change low (A1, B1, C0) | 2 | 1 | 0 |
| 42 | Dementia with Lewy bodies (brainstem-predominant) | 1* | 2 | 1 |
| 43 | Normal neuropathological examination | 0* | 1 | 0 |
| 44 | Alzheimer disease neuropathologic change low (A1, B2, C1) | 0 | 2 | 1 |
| 45 | Parkinson's disease (Braak 4) | 2* | 1 | 0 |
| 46 | Alzheimer disease neuropathologic change low (A1, B1, C1) | 0 | 2 | 1 |
| 47 | Alzheimer disease neuropathologic change intermediate (A2, B2, C2) | 0 | 2 | 0 |
| 48 | Normal neuropathological examination | 0* | 0 | 0 |
| 49 | Normal neuropathological examination with T2DM | 1* | 2 | 0 |
| 50 | Incidental Lewy bodies disease (Braak 1) | 0* | 1 | 1 |
| 51 | Normal neuropathological examination with T2DM | 2* | 2 | 1 |
| 52 | Normal neuropathological examination | 0* | 2 | 0 |
| 53 | Alzheimer disease neuropathologic change intermediate (A2, B3, C1) | 3 | 3 | 1 |
| 54 | Normal neuropathological examination | 0* | 2 | 0 |
| 55 | Alzheimer disease neuropathologic change intermediate (A3, B2, C2) | 3 | 2 | 1 |
| 56 | Alzheimer disease neuropathologic change intermediate (A2, B3, C2) | 0 | 2 | 0 |
| 57 | Alzheimer disease neuropathologic change intermediate (A2, B3, C1) | 2 | 1 | 1 |
| 58 | Alzheimer disease neuropathologic change intermediate (A2, B2, C2) | 0 | 1 | 0 |
| 59 | Normal neuropathological examination | 0* | 0 | 0 |
| 60 | Alzheimer disease neuropathologic change low (A1, B2, C1) | 0 | 0 | 0 |
| 61 | Alzheimer disease neuropathologic change intermediate (A2, B3, C2) | 0 | 0 | 0 |
| 62 | Alzheimer disease neuropathologic change low (A1, B1, C1) | 1 | 2 | 0 |
| 63 | Normal neuropathological examination with T2DM | 1* | 2 | 0 |
| 64 | Alzheimer disease neuropathologic change intermediate (A2, B2, C1) | 0 | 2 | 0 |
| 65 | Alzheimer disease neuropathologic change intermediate (A2, B2, C1) | 0 | 2 | 0 |
| 66 | Normal neuropathological examination with T2DM | 1* | 1 | 0 |
| 67 | Alzheimer disease neuropathologic change low (A1, B1, C1) | 2 | 2 | 0 |
| 68 | Alzheimer disease neuropathologic change low (A1, B3, C1) | 2 | 2 | 0 |
| 69 | Normal neuropathological examination with T2DM | 1* | 2 | 1 |
| 70 | Alzheimer disease neuropathologic change low (A1, B2, C1) | 0 | 0 | 0 |
| 71 | Normal neuropathological examination | 0* | 0 | 0 |
| 72 | Alzheimer disease neuropathologic change low (A1, B1, C1) | 0 | 2 | 0 |
| 73 | Alzheimer disease neuropathologic change low (A2, B1, C1) | 1 | 2 | 0 |
| 74 | Alzheimer disease neuropathologic change intermediate (A2, B3, C2) | 0 | 1 | 0 |
| 75 | Alzheimer disease neuropathologic change intermediate (A1, B2, C2) | 0 | 2 | 0 |
| 76 | Normal neuropathological examination | 0* | 2 | 1 |
| 77 | Normal neuropathological examination | 0* | 0 | 0 |
| 78 | Normal neuropathological examination | 0* | 0 | 0 |
| 79 | Normal neuropathological examination | 0* | 0 | 0 |
| 80 | Alzheimer disease neuropathologic change low (A1, B1, C1) | 0 | 2 | 0 |
| 81 | Normal neuropathological examination | 0* | 0 | 0 |
| 82 | Normal neuropathological examination | 0* | 0 | 0 |
| 83 | Alzheimer disease neuropathologic change low (A1, B2, C1) | 0 | 2 | 0 |
| 84 | Normal neuropathological examination with T2DM | 2* | 2 | 0 |
| 85 | Parkinson's disease (Braak 3) | 1* | 2 | 1 |
| 86 | Alzheimer disease neuropathologic change intermediate (A2, B2, C2) | 1 | 2 | 0 |
| 87 | Normal neuropathological examination | 1* | 2 | 1 |
| 88 | Normal neuropathological examination with T2DM | 2* | 2 | 2 |
| 89 | Normal neuropathological examination with T2DM | 2* | 3 | 1 |
| 90 | Dementia with Lewy bodies (brainstem-predominant) | 1* | 2 | 1 |
| 91 | Alzheimer disease neuropathologic change low (A1, B2, C1) | 2 | 1 | 1 |
| 92 | Alzheimer disease neuropathologic change low (A1, B2, C1) | 3 | 3 | 1 |
| 93 | Dementia with Lewy bodies (brainstem-predominant) | 1* | 2 | 0 |
| 94 | Normal neuropathological examination | 2* | 3 | 1 |
| 95 | Incidental Lewy bodies disease (Braak 1) | 1* | 2 | 0 |
| 96 | Alzheimer disease neuropathologic change intermediate (A2, B3, C2) | 3 | 2 | 1 |
| 97 | Normal neuropathological examination with T2DM | 0* | 2 | 1 |
| 98 | Dementia with Lewy bodies (brainstem-predominant) | 0* | 2 | 0 |
| 99 | Dementia with Lewy bodies (brainstem-predominant) | 1* | 2 | 0 |
| 100 | Incidental Lewy bodies disease (Braak 1) | 0* | 2 | 0 |
| 101 | Alzheimer disease neuropathologic change intermediate (A2, B3, C3) | 2 | 2 | 1 |
| 102 | Normal neuropathological examination with T2DM | 2* | 1 | 0 |
| 103 | Parkinson's disease (Braak 3) | 1* | 2 | 1 |
| 104 | Normal neuropathological examination with T2DM | 2* | 3 | 1 |
| 105 | Alzheimer disease neuropathologic change high (A3, B3, C3) | 1 | 1 | 0 |
| 106 | Normal neuropathological examination | 0* | 0 | 0 |
| 107 | Normal neuropathological examination with T2DM | 0* | 2 | 0 |
| 108 | Incidental Lewy bodies disease (Braak 1) | 0* | 2 | 0 |
| 109 | Normal neuropathological examination | 0* | 2 | 0 |
| 110 | Alzheimer disease neuropathologic change low (A1, B2, C1) | 0 | 2 | 0 |
| 111 | Alzheimer disease neuropathologic change low (A1, B3, C1) | 2 | 2 | 1 |
| 112 | Normal neuropathological examination with T2DM | 0* | 1 | 0 |
| 113 | Dementia with Lewy bodies (brainstem-predominant) | 2* | 3 | 1 |
| 114 | Alzheimer disease neuropathologic change intermediate (A1, B2, C1) | 0 | 2 | 0 |
| 115 | Alzheimer disease neuropathologic change high (A3, B3, C3) | 1 | 3 | 0 |
| 116 | Alzheimer disease neuropathologic change low (A1, B2, C1) | 0 | 2 | 1 |
| 117 | Normal neuropathological examination | 0* | 2 | 0 |
| 118 | Normal neuropathological examination with T2DM | 0* | 2 | 0 |
| 119 | Alzheimer disease neuropathologic change low (A1, B2, C1) | 0 | 2 | 0 |
| 120 | Alzheimer disease neuropathologic change intermediate (A2, B2, C2) | 0 | 2 | 0 |
| 121 | Alzheimer disease neuropathologic change low (A1, B1, C1) | 0 | 0 | 0 |
| 122 | Alzheimer disease neuropathologic change low (A1, B1, C1) | 2 | 2 | 1 |
| 123 | Alzheimer disease neuropathologic change high (A3, B3, C3) | 0 | 2 | 0 |
| 124 | Dementia with Lewy bodies (brainstem-predominant) | 2* | 3 | 1 |
| 125 | Parkinson's disease (Braak 3) | 0* | 2 | 1 |
| 126 | Alzheimer disease neuropathologic change low (A1, B1, C0) | 0 | 0 | 0 |
| 127 | Normal neuropathological examination | 0* | 0 | 0 |
| 128 | Parkinson's disease (Braak 4) | 1* | 2 | 0 |
| 129 | Alzheimer disease neuropathologic change low (A1, B2, C1) | 0 | 0 | 0 |
| 130 | Alzheimer disease neuropathologic change intermediate (A2, B2, C2) | 3 | 3 | 2 |
| 131 | Dementia with Lewy bodies (limbic) | 3* | 2 | 0 |
| 132 | Incidental Lewy bodies disease (Braak 2) | 2* | 3 | 0 |
| 133 | Incidental Lewy bodies disease (Braak 2) | 3* | 3 | 1 |
| 134 | Incidental Lewy bodies disease (Braak 2) | 3* | 3 | 1 |
| 135 | Incidental Lewy bodies disease (Braak 2) | 1* | 2 | 0 |
| 136 | Alzheimer disease neuropathologic change intermediate (A2, B2, C2) | 0 | 2 | 1 |
| 137 | Parkinson's disease (Braak 3-4) | 2* | 3 | 0 |
| 138 | Dementia with Lewy bodies (brainstem-predominant) | 3* | 3 | 1 |

**Table S4.** Scores obtained by subjects with Parkinson’s disease, dementia with Lewy bodies and incidental Lewy bodies disease in the semi-quantitative evaluation of Aβ and tau expression in pancreatic β-cells. Tau AT-8, Ser202-Thr205 phosphorylated tau; Tau AT-100, Thr212-Ser214 phosphorylated tau; Tau AT-180, Thr231 phosphorylated tau; Tau AT-270, Thr181 phosphorylated tau; pTau, phosphorylated tau.

| **ID** | **Neuropathological diagnosis** | **Scores** | | | | | | | | | | |
| --- | --- | --- | --- | --- | --- | --- | --- | --- | --- | --- | --- | --- |
|  |  | **AT-8** | **AT-100** | **AT-180** | **AT-270** | **pSer 422** | **pSer 262** | **Oligomeric tau** | **Tau cleaved** | **Alz-50** | **MC-1** | **Aβ (6E10)** |
| 1 | Parkinson's disease (Braak 4) | 3 | 1 | 0 | 1 | 1 | 1 | 0 | 2 | 2 | 1 | 0 |
| 4 | Parkinson's disease (Braak 3) | 3 | 2 | 0 | 2 | 1 | 3 | 1 | 2 | 3 | 1 | 1 |
| 5 | Dementia with Lewy bodies (brainstem-predominant) | 2 | 2 | 0 | 1 | 1 | 3 | 0 | 2 | 1 | 1 | 0 |
| 8 | Dementia with Lewy bodies (brainstem-predominant) | 3 | 2 | 2 | 2 | 2 | 2 | 0 | 2 | 2 | 0 | 0 |
| 9 | Parkinson's disease (Braak 4) | 3 | 1 | 0 | 1 | 1 | 3 | 1 | 2 | 3 | 0 | 0 |
| 15 | Dementia with Lewy bodies (brainstem-predominant) | 3 | 2 | 2 | 2 | 2 | 2 | 1 | 2 | 1 | 0 | 1 |
| 16 | Parkinson's disease (Braak 4) | 3 | 1 | 1 | 1 | 1 | 2 | 1 | 2 | 1 | 0 | 0 |
| 42 | Dementia with Lewy bodies (brainstem-predominant) | 2 | 3 | 1 | 2 | 3 | 3 | 3 | 2 | 2 | 1 | 1 |
| 45 | Parkinson's disease (Braak 4) | 3 | 1 | 1 | 2 | 2 | 2 | 2 | 2 | 1 | 0 | 0 |
| 50 | Incidental Lewy bodies disease (Braak 1) | 2 | 0 | 0 | 2 | 0 | 2 | 0 | 1 | 2 | 1 | 1 |
| 85 | Parkinson's disease (Braak 3) | 3 | 2 | 1 | 1 | 1 | 2 | 1 | 1 | 2 | 0 | 0 |
| 90 | Dementia with Lewy bodies (brainstem-predominant) | 1 | 1 | 0 | 1 | 0 | 2 | 1 | 0 | 1 | 0 | 0 |
| 93 | Dementia with Lewy bodies (brainstem-predominant) | 1 | 2 | 1 | 1 | 1 | 2 | 1 | 0 | 1 | 0 | 1 |
| 95 | Incidental Lewy bodies disease (Braak 1) | 2 | 1 | 0 | 2 | 1 | 1 | 0 | 0 | 0 | 0 | 0 |
| 98 | Dementia with Lewy bodies (brainstem-predominant) | 1 | 1 | 0 | 2 | 2 | 1 | 2 | 2 | 1 | 0 | 0 |
| 99 | Dementia with Lewy bodies (brainstem-predominant) | 3 | 1 | 1 | 2 | 0 | 2 | 1 | 1 | 2 | 1 | 0 |
| 100 | Incidental Lewy bodies disease (Braak 1) | 1 | 1 | 0 | 2 | 0 | 1 | 2 | 0 | 0 | 0 | 1 |
| 103 | Parkinson's disease (Braak 3) | 2 | 2 | 0 | 2 | 1 | 2 | 1 | 0 | 1 | 0 | 1 |
| 108 | Incidental Lewy bodies disease (Braak 1) | 2 | 1 | 0 | 1 | 1 | 1 | 2 | 2 | 2 | 0 | 0 |
| 113 | Dementia with Lewy bodies (brainstem-predominant) | 2 | 1 | 0 | 1 | 1 | 1 | 2 | 1 | 2 | 1 | 2 |
| 124 | Dementia with Lewy bodies (brainstem-predominant) | 2 | 2 | 0 | 1 | 2 | 2 | 3 | 1 | 2 | 0 | 0 |
| 125 | Parkinson's disease (Braak 3) | 3 | 0 | 0 | 1 | 0 | 1 | 1 | 0 | 2 | 1 | 1 |
| 128 | Parkinson's disease (Braak 4) | 2 | 2 | 0 | 1 | 0 | 1 | 0 | 1 | 2 | 0 | 0 |
| 131 | Dementia with Lewy bodies (limbic) | 3 | 1 | 2 | 2 | 2 | 2 | 2 | 1 | 2 | 2 | 0 |
| 132 | Incidental Lewy bodies disease (Braak 2) | 1 | 0 | 0 | 2 | 1 | 3 | 1 | 2 | 1 | 0 | 0 |
| 133 | Incidental Lewy bodies disease (Braak 2) | 2 | 2 | 1 | 3 | 1 | 3 | 1 | 3 | 1 | 1 | 0 |
| 134 | Incidental Lewy bodies disease (Braak 2) | 1 | 1 | 0 | 3 | 1 | 2 | 1 | 2 | 1 | 0 | 0 |
| 135 | Incidental Lewy bodies disease (Braak 2) | 0 | 1 | 0 | 2 | 0 | 2 | 1 | 2 | 1 | 0 | 0 |
| 137 | Parkinson's disease (Braak 3-4) | 1 | 1 | 1 | 2 | 1 | 2 | 1 | 2 | 1 | 1 | 0 |
| 138 | Dementia with Lewy bodies (brainstem-predominant) | 3 | 2 | 2 | 2 | 1 | 2 | 0 | 1 | 1 | 0 | 0 |

**Figures**

**Figure S1.** Immunohistochemistry for Aβ (a, b) and total tau (c-f) in the gastric mucosa of pancreatic cells from a 75-year-old male with Parkinson’s disease and no history of T2DM. Negative control of the in situ PLA assay for PrP and tau (g, h), and PrP and amylin (i, j), where no anti-PrP primary antibody was added. DAPI nuclear counterstaining was used; a, c, e 20x magnification; b, d, f, g-j 40 x magnification; scale bar = 150 µm.


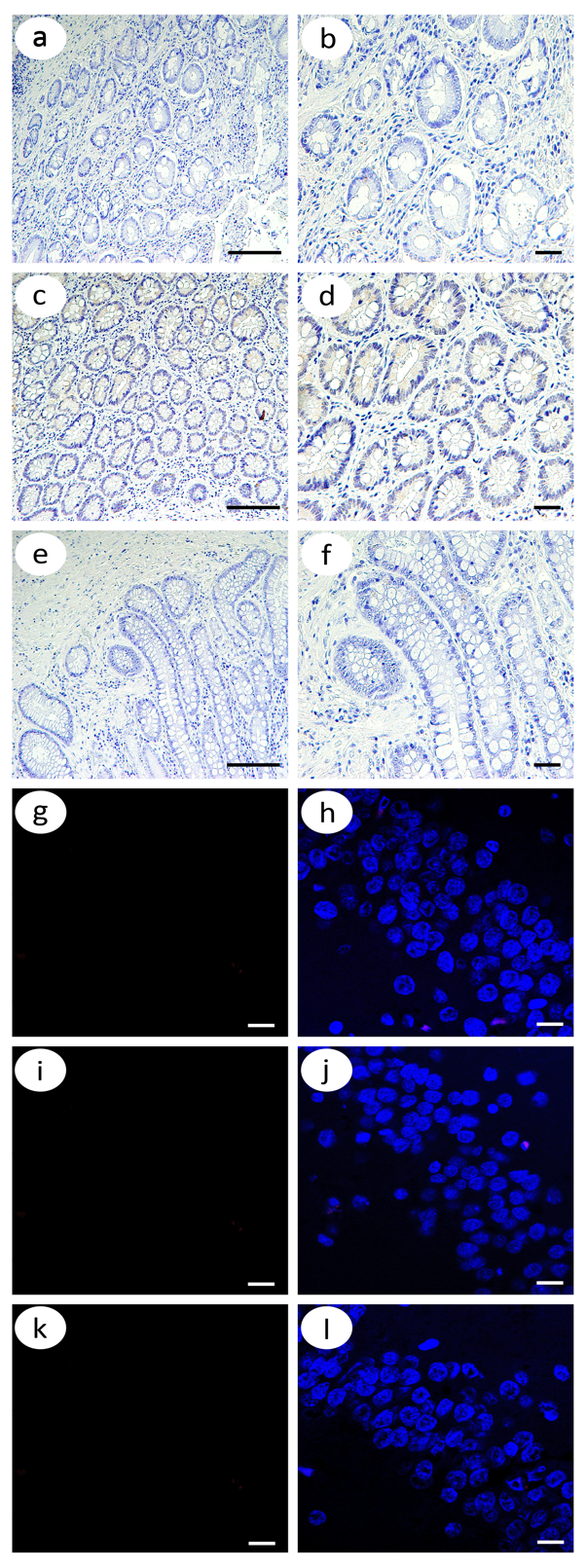


**Figure S2.** Score matrix used for the semi-quantitative evaluation of protein expression in the pancreas.


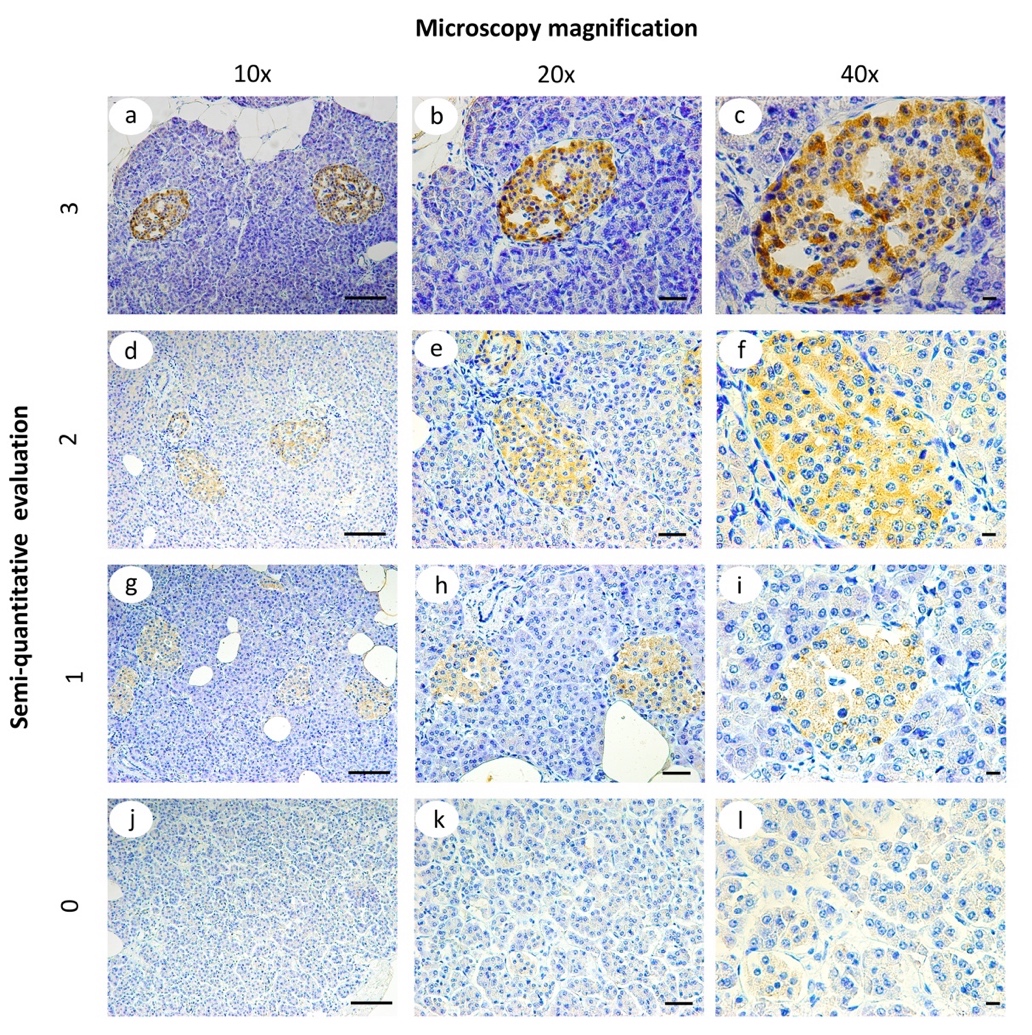


**Figure S3.** PLA assay for PrP and chromogranin A (a-c), amylin and chromogranin A (d-f) and tau with chromogranin A (g-i) with insulin immunofluorescence (b, e, h) in the pancreas from a 73-year-old female with Alzheimer’s disease. PLA assay for PrP and amylin (j-k) and PrP and phosphorylated alpha-synuclein (l-m) in the *locus coeruleus* from a 60 years-old female with a normal neuropathological examination. DAPI nuclear counterstaining was used; 40 x magnification; scale bar = 150 µm.


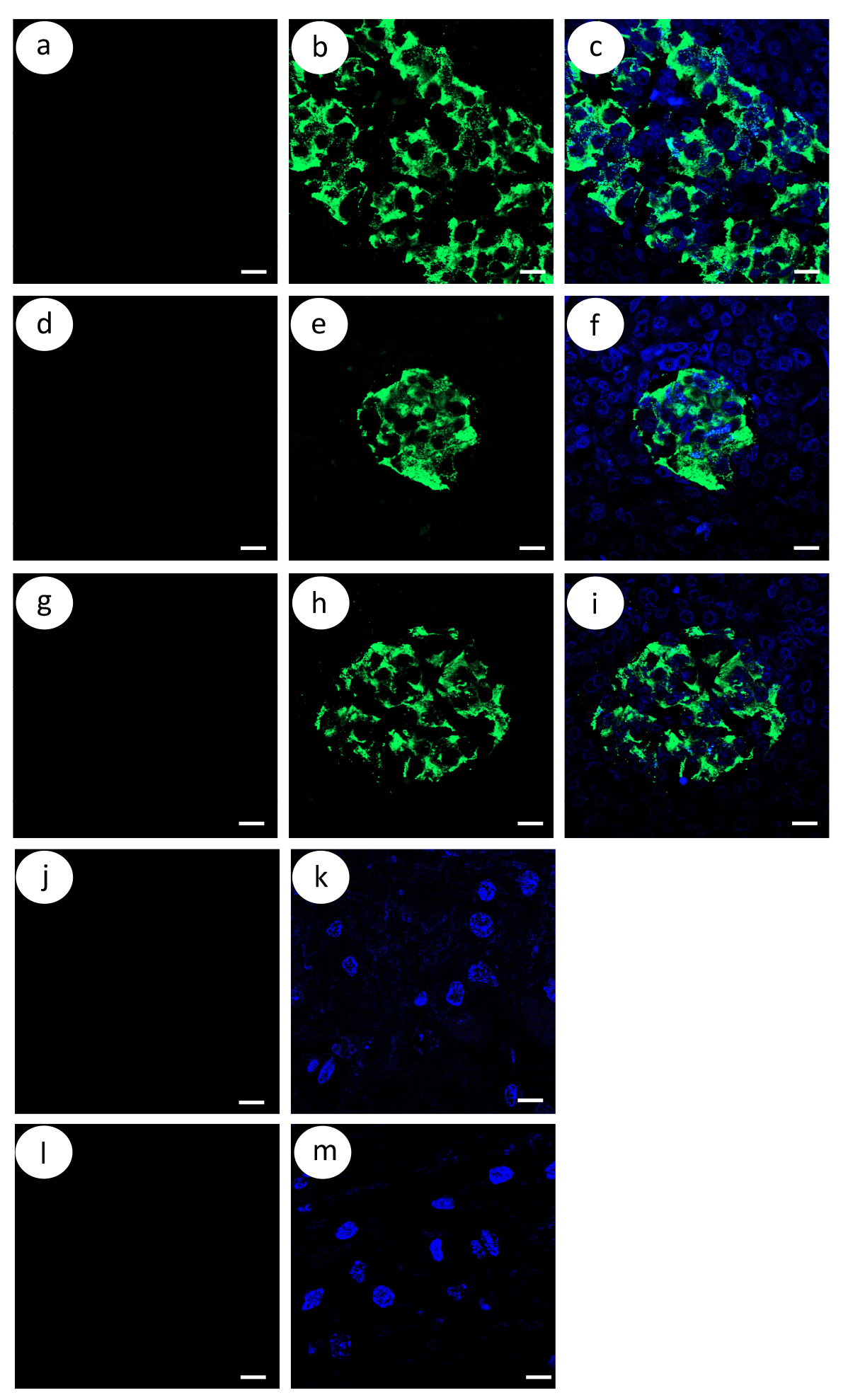


**Figure S4.** Immunohistochemistry for amylin (a), α-synuclein (b), tau (c) and PrP (d) in the pancreatic tissue from a 79-year-old male with AD. 4x magnification; scale bar = 600µm.


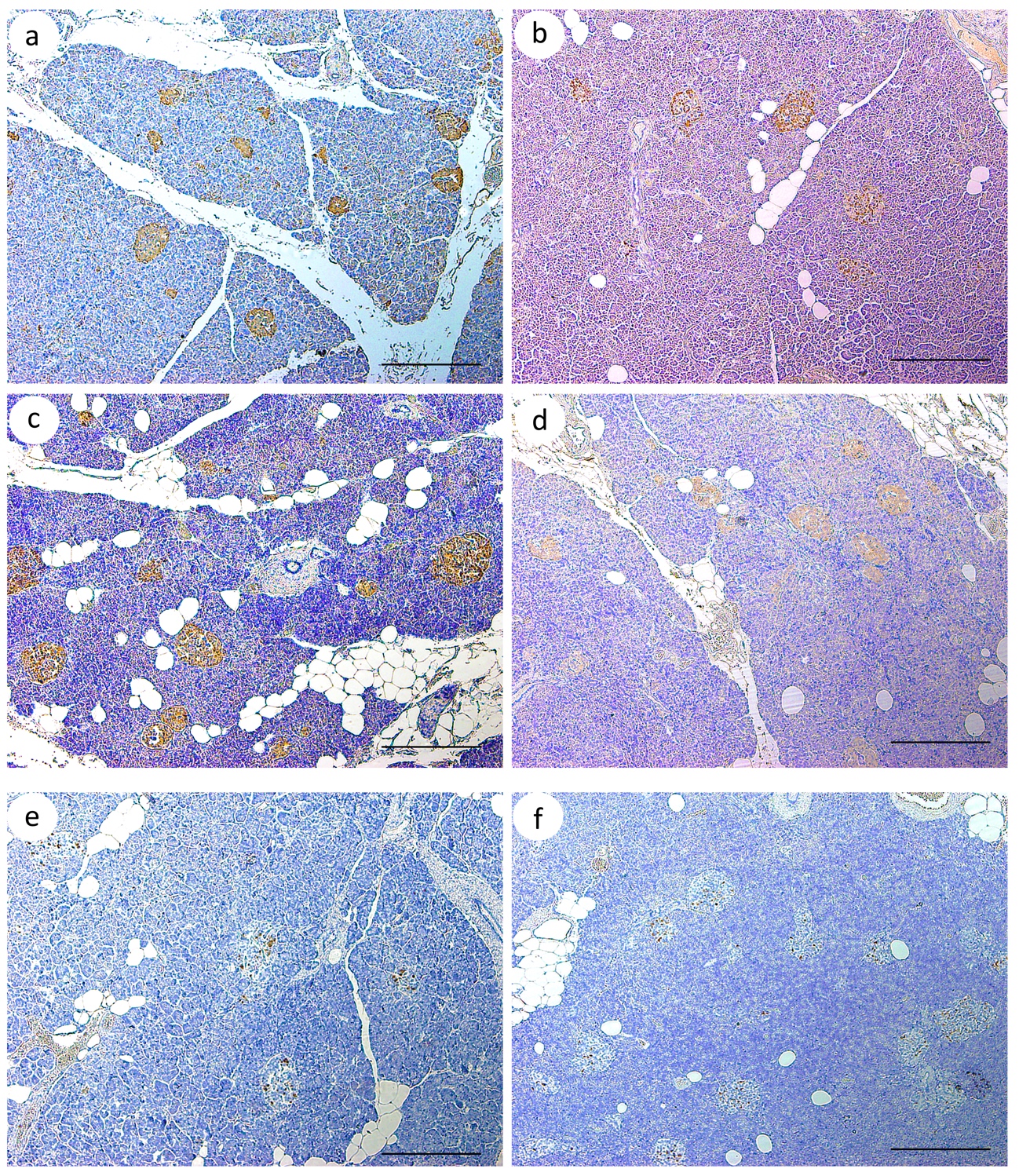


**Figure S5**. Immunohistochemistry for oligomeric tau (T22) in the pancreatic tissue from a 77-year-old female with Parkinson’s disease (a-c), and from a 69-year-old female with a normal neuropathological examination without type two diabetes mellitus (d-f). Figure (a, d) 10x magnification; figure (b, e) 20 x magnification; figure (c, f) 20 x magnification scale bar = 150µm.


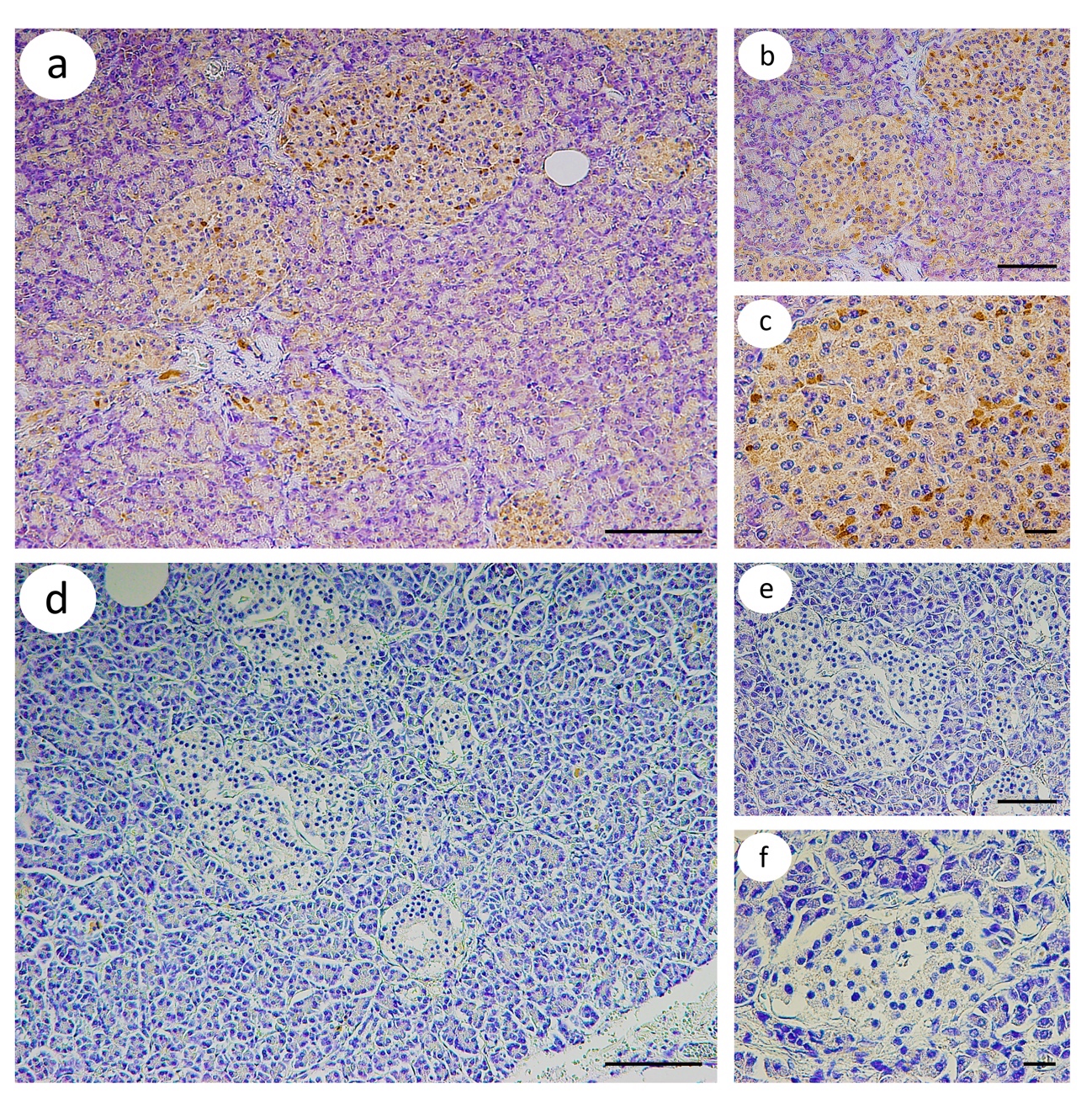


**Figure S6**. Tau immunofluorescence (a) and thioflavin S fluorescence (b) in pancreatic β cells from a 77-year-old female with Alzheimer’s disease (c). Dual immunofluorescence for tau and glucagon (d-f), somatostatin (g-i) or insulin (j-l) to assess the cellular localization of tau in the pancreas from a 73-year-old female with Parkinson’s disease: 40x magnification; scale bar = 50 µm.


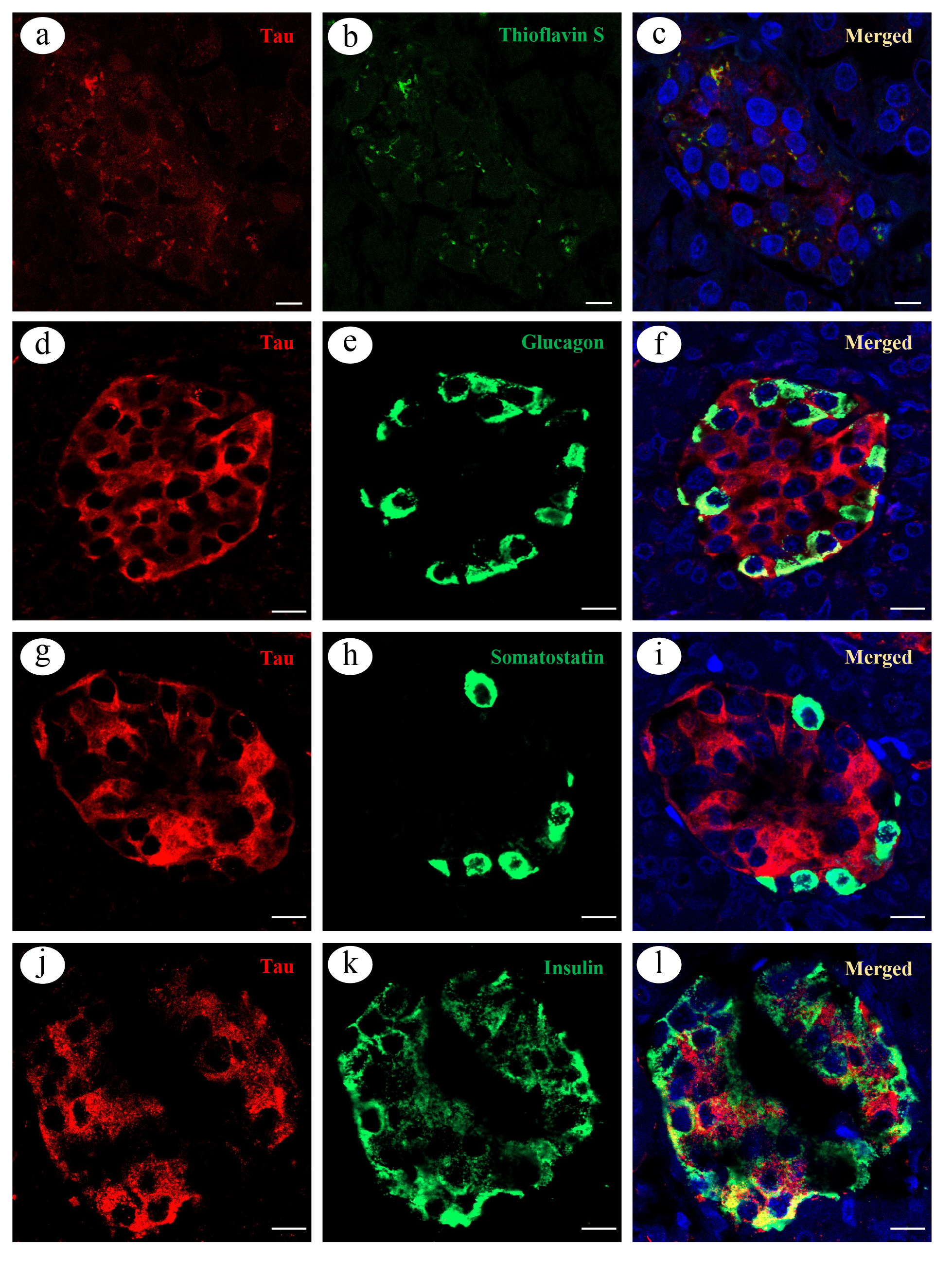


**Figure S7**. Dual immunofluorescence for amyloid beta and anti-oligomeric A11 antibody (a-c) and amyloid beta with insulin (d-f) from a 73-year-old female with Parkinson’s disease: 40x magnification; scale bar = 50 µm.


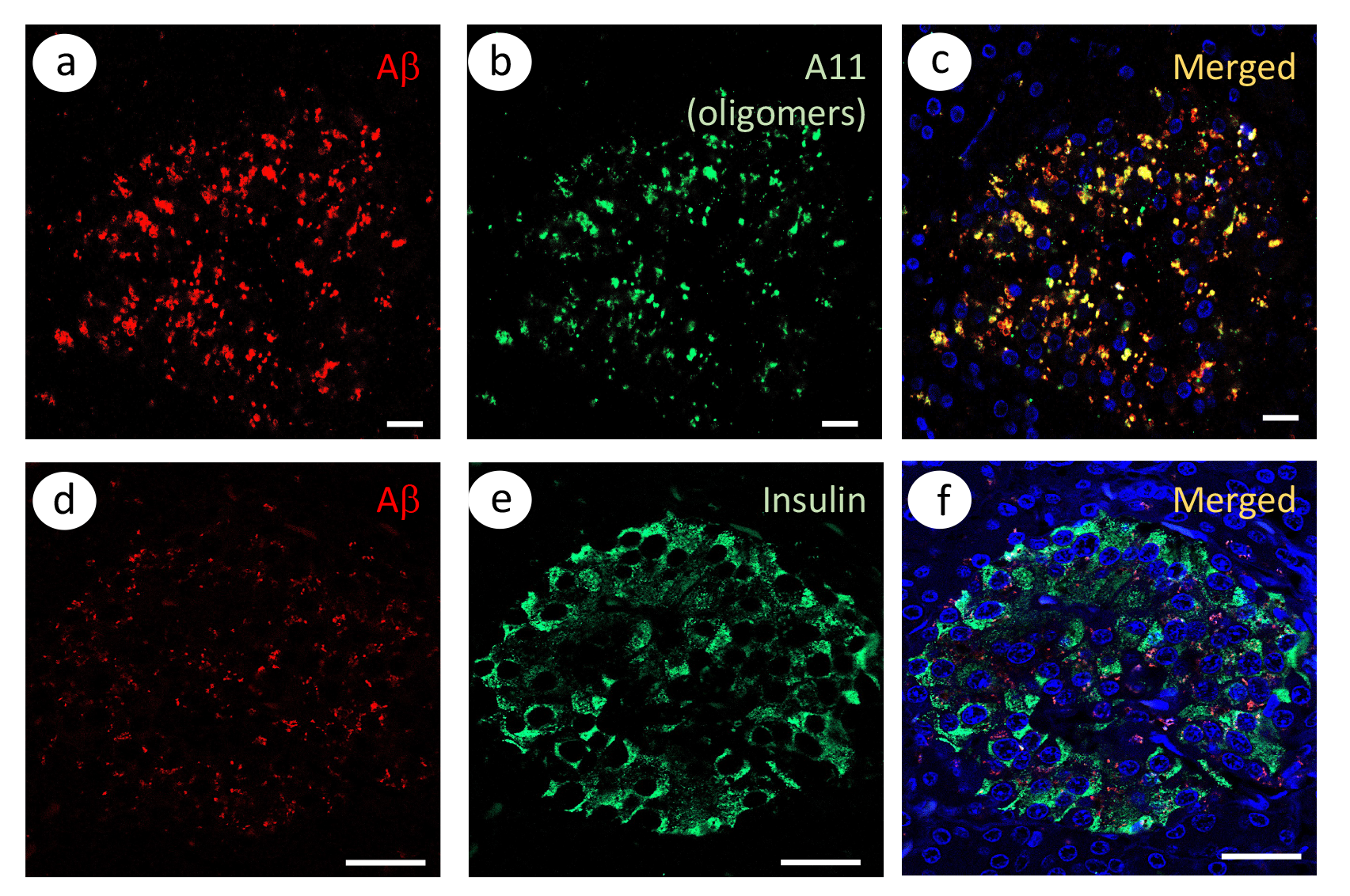

Supplement: Supplementary file 1 — Additional file 1. Supplementary figures and tables. [file 40478_2021_1171_MOESM1_ESM.docx]
